# Strategies to self-manage side-effects of adjuvant endocrine therapy among breast cancer survivors: an umbrella review of empirical evidence and clinical guidelines

**DOI:** 10.1007/s11764-021-01114-7

**Published:** 2021-10-18

**Authors:** Louise H. Hall, Natalie V. King, Christopher D. Graham, Sophie M. C. Green, Alice Barber, Richard D. Neal, Robbie Foy, Jane Clark, Kelly E. Lloyd, Samuel G. Smith

**Affiliations:** 1 Leeds Institute of Health Sciences, University of Leeds, Leeds, LS2 9NL England, UK; 2 Department of Psychology, Queen’s University Belfast, Belfast, BT7 1NN Northern Ireland UK; 3 School of Medicine, University of Leeds, Leeds, LS2 9NL England, UK; 4 Department of Clinical and Health Psychology, St James’s University Hospital, Leeds, LS9 7TF England, UK

**Keywords:** Breast cancer, Adjuvant endocrine therapy, Complementary therapy, Self-management, Systematic review, Side-effects

## Abstract

**Purpose:**

Side-effects of adjuvant endocrine therapy (AET) are common in breast cancer survivors, and can affect adherence to treatment. We synthesised the evidence for strategies to self-manage these side-effects.

**Methods:**

We searched for systematic reviews and clinical guidelines on self-management strategies for AET side-effects (arthralgia, fatigue, hot flashes, gastrointestinal discomfort, nausea, vulvovaginal symptoms, and sleep disturbance). We searched oncology organisation’s websites and eight databases (Inception-November 2020). Screening, data extraction and quality assessment were completed independently in duplicate. PROSPERO: 2019CRD4201914001.

**Results:**

We identified 33 systematic reviews and 18 clinical guidelines. 21% of reviews were high quality, and the average quality score for guidelines was 44%. Evidence for most strategies was absent or weak. There was consensus from a low-quality review and multiple guidelines to recommend moisturisers, gels and lubricants for vulvovaginal symptoms. Evidence was weak for physical activity for self-managing most symptoms, although two high-quality reviews indicated yoga and aerobic exercise could reduce fatigue. Primary research was often biased by weak and underpowered study designs. Eleven reviews did not report information on adverse events.

**Conclusions:**

Most self-management strategies for breast cancer survivors experiencing side-effects from AET lack evidence. Primary research is needed using high-quality well-powered designs focusing on implementable strategies.

**Implications for Cancer Survivors:**

Patients and clinicians should be aware that although the risk of harm is low for these self-management strategies, the likelihood of benefit is often unclear. Women should consider moisturisers, gels or lubricants for self-managing vulvovaginal symptoms, and yoga or aerobic exercise for alleviating fatigue.

**Supplementary Information:**

The online version contains supplementary material available at 10.1007/s11764-021-01114-7.

## Introduction

Breast cancer is the most common cancer in women worldwide, and the leading cause of cancer death [1]. Most tumours are oestrogen-receptor positive (ER +), which can be treated in the adjuvant setting with endocrine therapies. These include tamoxifen and Aromatase Inhibitors (AIs), such as letrozole, anastrozole, and exemestane. Common side-effects reported by tamoxifen users include hot flashes, fatigue and vulvovaginal symptoms, while arthralgia is commonly reported in women using AIs [2–6]. Most women experiencing these symptoms want more support to manage them [6]. Experiencing these symptoms can reduce quality of life [2, 7–11], and is likely to contribute to the estimated three-quarters of women who struggle to adhere to and persist with adjuvant endocrine therapy (AET) [12]. Patients and oncologists perceive side-effects to be a major deterrent to AET adherence [13, 14].

Women with breast cancer who do not adhere to AET have an increased risk of all-cause and cancer-specific mortality [15–19] and recurrence [16, 20, 21]. It is therefore important to provide evidence-based support to women experiencing side-effects from AET. Women often self-manage these side-effects [6], but use a wide range of strategies which may not be evidence-based. There is a large volume of scientific research and clinical guidelines available on this topic. Therefore, an overall examination of this body of information is needed to identify consistencies and discrepancies in recommendations, and to provide patients and healthcare professionals with clear evidence-based advice for managing these symptoms [22]. We undertook a systematic review of systematic reviews and clinical guidelines (also called an umbrella review) to synthesise evidence for self-management strategies for side effects among women using AET. This approach provides a method for summarising a broad topic of related issues, thereby providing a single source of information for patients, healthcare professionals and research scientists. The inclusion of clinical guidelines within the review will enable us to observe the extent to which evidence is being translated into clinical practice recommendations, to identify self-management strategies recommended by clinical guidelines that are not supported by evidence, and to assess the quality of guidelines informing clinical care.

## Methods

We were interested in literature related to individual-level non-pharmacological or lifestyle treatments or management strategies that could be accessed without seeking a prescription or referral from a healthcare professional. These interventions could be compared against no intervention, usual care or other strategies or treatments, including those administered by a healthcare professional. We were interested in systematic reviews and clinical guidelines reporting outcome measures assessing AET side-effects. We created a list of all side-effects associated with tamoxifen, letrozole, anastrozole, and exemestane and presented them to two primary care physicians (RF and RN). Consensus was reached that the following could be safely managed without initially seeking support from a healthcare professional: arthralgia (including joint pain and joint disorders), gastrointestinal discomfort, fatigue, hot flashes, nausea, vulvovaginal symptoms, and sleep disturbance.

The review was prospectively pre-registered (PROSPERO 2019 CRD4201914001). We adhered to the PRISMA 2020 checklist throughout [23].

### Search strategy

In March 2019, we searched for systematic reviews examining the self-management strategies of common side effects experienced by breast cancer patients being treated with AETs. We updated the searches in November 2020. The following databases were searched from inception to present; CINAHL, Cochrane, Embase, Medline, and Web of Science (Appendix). The database searches were developed for three search concepts: breast cancer; tamoxifen or Aromatase Inhibitors and generic and specific side effects. Database subject headings and free text words were identified by the Information Specialist (NK) and project team members (SS, LH). The search was peer-reviewed by another Information Specialist using the PRESS checklist [24]. To identify relevant clinical guidelines, we searched for AETs and the named side-effects in the following websites in July 2019 and November 2020: American Society for Clinical Oncology (ASCO), European Society of Medical Oncology (ESMO), NIH National Cancer Institute (NCI), National Comprehensive Cancer Network (NCCN), Scottish Intercollegiate Guidelines Network (SIGN), and National Institute for Health and Care Excellence (NICE) Evidence. We also searched the guidelines databases; Turning Research Into Practice (TRIP), International Guidelines Library (GIN), and National Health and Medical Research Council (NHMRC) Australian Clinical Practice Guidelines. The results were stored and deduplicated using Endnote X9 software. Further relevant studies were sought by backward citation searching of the included studies.

### Study selection

Screening was conducted in two stages using Covidence software. Two authors (SS, LH, or ER) independently screened the titles and abstracts for eligibility and conducted the full text screen. Disagreements were resolved through discussion with a third author (CG). We included systematic reviews and meta-analyses and guidelines from societies, government agencies and charities with the following criteria; women with breast cancer using AET (e.g. tamoxifen, aromatase inhibitors, anastrozole, letrozole or exemestane). The interventions were individual-level, non-pharmacological or lifestyle treatments or management strategies available without a prescription or referral from healthcare professionals. They could be compared against usual care, other self-management strategies, or treatments administered by a healthcare professional. We excluded narrative reviews and studies reporting primary data, reviews focussed mainly on cancers other than breast cancer, symptoms not associated with AET, switching treatments, the neoadjuvant setting or side-effects that require clinical management.

### Data collection

Data extraction was conducted in Microsoft Excel in duplicate (SS, LH), with a third author (KL) arbitrating any disagreements. The data extraction form was piloted before full data extraction took place. Key study variables extracted for the systematic reviews are shown in Table 1. We also extracted information on study designs eligible, approach to synthesis, self-management strategies reported, sample size and effect sizes in meta-analysis, key findings and limitations. Key study variables extracted for the clinical guidelines are shown in Table 2.

**Table 1:** Characteristics of systematic reviews investigating self-management strategies for side-effects among women using AET

Author / date	Objectives	Review type	Participants (n)	Databases searched (date range)	Relevant articles	Country of origin	Eligible study designs	Quality appraisal	Outcomes / side-effect	AMSTAR2 score	Adverse events summary
Bae et al., 2015 [31]	To evaluate published studies reporting the effect of acupuncture on AI-induced arthralgia	Systematic review	Breast cancer (n = 193)	PubMed, Medline, ProQuest, ScienceDirect, Cochrane Library, EMBASE, CINAHL, PsychInfo (all inception-May 2014)	4	US, Australia	RCTs	Cochrane Risk of Bias Tool	Arthralgia	Low	No severe events. Some minor bruising and pain
Chao et al., 2009 [32]	To scrutinize the evidence on the use of acupoint stimulation for managing therapy-related adverse events in breast cancer	Systematic review and meta-analysis	Breast cancer any stage (n = 281)	PubMed, Cochrane Library, EMBASE, CINAHL, PsychInfo, CNKI, CEPS, Wangang (all inception-October 2008)	8	US, China, UK, Sweden, Other	RCTs, single-group studies	Modified Jadad	Arthralgia, hot flashes	Low	Most studies did not report. Minor bleeding and itchiness in 2 studies
Chen et al., 2017 [40]	To investigate the effectiveness of acupuncture approach in relieving aromatase inhibitor-induced arthralgia in breast cancer patients	Systematic review and meta-analysis	Breast cancer, stage 1–3 using AIs for more than 1 month (n = 181)	Pubmed, Embase, Scopus, Cochrane Library; clinicaltrials.gov (all inception-2017)	5	US and Australia	RCTs	Cochrane Risk of Bias Tool	Arthralgia	Moderate	2 studies reported 18 minor events (tingling, numbness)
Chien et al., 2015 [41]	To summarize and critically assess the evidence from RCTs that examined acupuncture in the treatment of AI associated muskuloskeletal symptoms in patients with breast cancer	Systematic review and meta-analysis	Breast cancer, stage 1–3, joint symptoms after taking AI (n = 207)	Medline, PubMed, EMBASE, Cochrane Library, CINAHL, PEDro, ITPLS, CNKI, WanFang, WHO ICTRP (all inception-February 2014)	5	US and Australia	RCTs	Modified Jadad	Arthralgia, pain	Moderate	No adverse events
Dowling et al., 2017 [28]	To determine the survivorship issues for postmenopausal women with breast cancer undergoing endocrine therapy, with a particular focus on arthralgia	Systematic review and meta-analysis	Breast cancer, > 45 years prescribed tamoxifen or AI (n = 368)	CINAHL, Cochrane Database of Systematic Reviews, EMBASE, Google Scholar, MEDLINE, PsychINFO, PubMed, Scopus, Sociological Abstracts (ProQuest) (all 2005–2015)	5	US, Australia and Spain	No exclusions	QATSDD	Arthralgia, fatigue	Critically low	No serious events for acupuncture of physical activity. Not reported for yoga
Fritz et al., 2013 [42]	To conduct a systematic review of soy and red clover for efficacy in improving menopausal symptoms in women with breast cancer	Systematic review	Women with and without breast cancer (n, not reported)	MEDLINE, EMBASE, AMED, Cochrane Library (Soy = Inception-March 2013; red clover = Inception-December 2012)	7	Not reported	Trials or observational	Cochrane Risk of Bias Tool	Hot flashes	Moderate	Gastrointestinal discomfort (soy) and minor events comparable between trial arms (red clover)
Fritz et al., 2014 [43]	To conduct a systematic review of black cohort for use by pre- and post-menopausal breast cancer patients, and to assess its efficacy in treating menopausal symptoms	Systematic review	Women with and without breast cancer (n = 1214)	MEDLINE, EMBASE, AMED, Cochrane Library, and trial registries (all inception-July 2012)	6	Germany, US, Venezuela	Trials or observational	Cochrane Risk of Bias Tool	Fatigue, hot flashes	Moderate	Mostly not reported. Some minor adverse events not attributed to black cohosh. 3 serious adverse events in women using black cohosh (breast cancer recurrence, hysterectomy)
Halsey et al., 2015 [27]	To determine the effectiveness and safety of acupuncture for the management of AI induced musculoskeletal syndrome in postmenopausal women with early-stage breast cancer	Systematic review	Breast cancer, stage 1–3 treated with AI and joint pain (n = 190)	AMED, CINAHL, Cochrane CENTRAL, Medline (all inception-January 2015)	4	US and Australia	RCTs	STRICTA guidelines and Cochrane Back Review Group tool	Arthralgia	Critically low	Minor pain and bruising in small proportion of patients
Johns et al., 2016 [53]	To report a systematic review comparing at least two non-hormonal hot flash treatments in breast cancer survivors	Systematic review	Breast cancer (n = 312)	Pubmed (1996-July 2015), SCOPUS (1966-July 2015), CINAHL (1981-July 2015), Cochrane (all years), Web of Science (1900-July 2015)	4	Egypt, Italy and US	Randomised trials	Cochrane Risk of Bias Tool	Hot flashes	High	Minor adverse events reported, but fewer than with pharmacological treatments
Kassab et al., 2009 [54]	To evaluate the effectiveness and safety of homeopathic medicines used to prevent or treat adverse effects associated with cancer treatments	Systematic review	Cancer (n = 136)	Cochrane pain & palliative care trials register (inception-November 2008), Cochrane Central Register of Controlled Trials (inception-November 2008), MEDLINE (1966–2008), EMBASE (1980–2008), CINAHL (1982–2008), BNI (1985–2008), Cancer LIT (1975–2008), AMED (1985–2008), CISCOM (1991-), Hom-Inform (1966-December 2009), SIGLE (1976–2003), National Research Register (1998-December 2007), Zetoc (1993-December 2008), www.controlled-trials.com (-December 2008), clinicaltrials.gov (-December 2008), Liga Medicorum Homeopathica Internationalis (LMHI, Liga)	2	UK and US	RCTs	Cochrane Risk of Bias Tool	Hot flashes	High	No serious events attributable to homeopathic medicine
Nahm et al., 2018 [47]	To systematically review strategies to manage arthralgia among women using AIs to treat breast cancer	Systematic review	Breast cancer, using Ais (n = 1349)	Medline, Cochrane, EMBASE (all inception to April 2015)	17	Not reported	No exclusions	Cochrane Risk of Bias Tool (adapted)	Arthralgia	Moderate	Not reported
Pan et al., 2015 [55]	To understand the potential benefit of Tai Chi Chuan in reducing treatment-related side-effects and quality of life in women with breast cancer	Systematic review and meta-analysis	Breast cancer (n = 207)	Pubmed (1966-Nov 2014), EMBASE (1974-Nov 2014), Cochrane Library (issue 11, 2014), Web of Science (1974-Nov 2014)	6	US	Randomised trials	Cochrane Risk of Bias Tool	Fatigue	High	Not reported
Pan et al., 2018 [44]	To assess the efficacy of acupuncture for treatment-related side-effects of hormone therapy and the quality of life of patients with breast cancer	Systematic review and meta-analysis	Breast cancer (n = 810)	Pubmed (1966-November 2017), EMBASE (1974-November 2017), Cochrane Library (issue 4–2017), Web of Science (1974-November 2017)	17	US, Sweden, Australia, Korea, Norway, Europe, UK	RCTs	Cochrane Risk of Bias Tool	Arthralgia, gastrointestinal, fatigue, hot flashes, pain, sleep disturbance	Moderate	No adverse events
Roberts et al., 2017 [33]	To summarise the recent literature on the symptom management intervention strategies for AIMSS	Systematic review and meta-analysis	Breast cancer, stage 1–3, adjuvant treatment with AI or at risk of AIMSS (n = 1631)	PubMed, EMBASE, CINAHL, CENTRAL, Google Scholar (all inception-February 2016)	26	US, Japan, Spain, China, Canada, England, Australia, Greece, France, Italy	RCTs, cohort and case–control	Jadad scale (RCTs) and Newcastle–Ottawa Scale (case control studies)	Arthralgia	Low	Not reported comprehensively, but described as minimal
Salehi et al., 2016 [34]	To evaluate the effectiveness of acupuncture for treatment of hot flash in women with breast cancer	Systematic review and meta-analysis	Breast cancer (n = 1361)	MEDLINE, CINAHL, EMBASE, Cochrane Breast Cancer Group Specialized Register, CENTRAL, DOAJ, Ovid, Wiley, Science Direct, EBSCO, Springer Link, and BMJ (all inception-2015)	12	Sweden, US, UK, Norway, Korea, Denmark	No exclusions	Modified Jadad scale	Hot flashes	Low	Not reported
Yang et al., 2017 [48]	To identify pain management of AI associated arthralgia and to evaluate the study quality and effects of interventions	Systematic review and meta-analysis	Breast cancer, stage 1–3 using an AI (n = 1076)	PubMed, CINAHL, PsychINFO, Web of Science, google scholar, ProQuest (all 2000-August, 2015)	15	US, Australia, UK, France, Japan, China	Randomised, quasi randomised, and prospective observational	Quality Assessment Tool for Quantitative Studies	Arthralgia	Moderate	Not reported
Garcia et al., 2015 [45]	To update findings from previous studies by 1) identifying randomised controlled trials evaluating acupuncture for hot flushes in cancer patients, 2) evaluating risk of bias for included studies on the basis of Cochrane criteria, and 3) calculating treatment effect size estimates	Systematic review	Cancer patients, with hot flashes (n = 474)	PubMed, Scopus, EMBASE, MEDLINE, CINAHL, Cochrane [all databases] (all inception-December 2014)	8	Not reported	RCTs	Cochrane Risk of Bias Tool	Hot flashes	Moderate	No adverse events
Lee et al., 2009 [46]	To summarize and critically assess the evidence from randomised controlled trials of acupuncture in treating hot flashes in breast cancer patients	Systematic review and meta-analysis	Breast cancer, with hot flashes (n = 281)	MEDLINE, CINAHL, EMBASE, PsychINFO, KSIKorean Studies Information, DBPIA, KISTI, Korea-Med, RICHB, CNKI, CJP, CMFD, CPCD, Cochrane Library 2008 Issue 3 (all inception-August 2008)	6	US, Sweden, UK, Norway	RCTs	Modified Jadad	Hot flashes	Moderate	Minor adverse events reported in two studies
Rada et al., 2010 [56]	To assess the efficacy of non-hormonal therapies in reducing hot flushes in women with a history of breast cancer	Systematic review and meta-analysis	Breast cancer, with hot flashes (n = 517)	Cochrane Breast Cancer Group Specialised Register (22 August 2008). Cochrane Library (2008, Issue 3), CINAHL, PsychoINFO, LILACS (inception to August, 2008), MEDLINE (January 1966-December 2005); EMBASE (1974 to April 2005), WHO ICTRP (May 2010)	7	US and UK	RCTs	Cochrane Risk of Bias Tool	Hot flashes	High	Inconsistently reported. No adverse events for vitamin E or homeopathy. Some minor bleeding and bruising with acupuncture
Tremblay et al., 2008 [35]	To assess the effects of psycho-educational interventions on hot flashes in menopausal women and women with a history of breast cancer	Systematic review	Cancer, with vasomotor symptoms (n = 138)	MEDLINE, CINAHL, PsycInfo, Cochrane Library (all 1980-December 2006)	3	Not reported	Any experimental	Newell criteria	Hot flashes	Low	Not reported
Cramer et al., 2017 [57]	To assess effects of yoga on health-related quality of life, mental health and cancer-related symptoms among women with a diagnosis of breast cancer who are receiving active treatment or have completed treatment	Systematic review and meta-analysis	Women with a histologically confirmed non-metastatic or metastatic breast cancer diagnosis (n = 1370)	Cochrane Breast Cancer Specialised Register, MEDLINE, Embase, CENTRAL; 2016, Issue 1), WHO ICTRP, Clinicaltrials.gov, IndMED all up to January 2016; and grey literature: ICCMR; ECIM; ASCO; all up to 2015	16	US, Germany, India, Turkey	RCTs	Cochrane Risk of Bias Tool	Fatigue, sleep disturbance	High	Not consistently reported. Generally small numbers of minor events, and no serious adverse events
Finnegan John et al., 2013 [36]	To provide a contemporary appraisal of the relative effectiveness of different CAM interventions in managing cancer-related fatigue	Systematic review	Adults with experience of treatment for cancer (n = 254)	CENTRAL, MEDLINE, EMBASE, BNI, PsychINFO, EBMR, AMED from inception to June 2012	5	Germany, UK, US, Korea	RCT or quasi-experimental	Oxford Quality Score / Jadad Score	Fatigue	Low	Not reported
Taylor et al., 2011 [37]	To explore the nature and effectiveness of interventions for treatment of sexual problems experienced by breast cancer patients	Systematic review	Women with breast cancer (n = 510)	Medline, Embase Classic and Embase, PsychINFO, CINAHL, CENTRAL from inception to January 2011	3	Not specified	No exclusions	Cochrane and Revenson quality grading system	Vulvovaginal symptoms, hot flashes	Low	Not reported
Cramp et al., 2012 [58]	To evaluate the effect of exercise on cancer-related fatigue both during and after cancer treatment	Systematic review and meta-analysis	Adults of any age experiencing cancer-related fatigue (n = 641)	CENTRAL (Issue 1, 2011), MEDLINE, EMBASE, CINAHL, BNI, AMED, SIGLE and DAI, from inception to March 2011)	13	Not specified	RCTs	Oxford Quality Score and Cochrane Risk of Bias tool	Fatigue	High	Not reported
Bordeleau et al., 2007 [29]	To systematically review the nonpharmacologic and pharmacologic options for the treatment of hot flashes in breast cancer survivors	Systematic review	Women with breast cancer (n = 779)	MEDLINE, EMBASE from 1990 to July 2006	7	US, Scotland, Canada, Finland	RCTs	Not used	Hot flashes	Critically low	Not reported
Mazzarello et al., 2015 [38]	To explore the evidence available for treatment options (both non-estrogen and estrogen-based) for urogenital atrophy in breast cancer patients	Systematic review	Women with breast cancer (n = 189)	EMBASE (1947-present), Ovid Medline (1946-present), Cochrane Library (November, 2014), and all abstracts since 2009	3	Not specified	RCTs	Cochrane Risk of Bias Tool	Vulvovaginal symptoms	Low	Mild irritation and burning with Ph gel, mild irritation, burning, itch and discharge with vaginal moisturiser
Ruan et al., 2019 [30]	To offer an overview of the experimental and clinical data on iCR/iCRþHP safety at the breast, its use in breast cancer patients, and putative interactions with endorcrine treatment	Systematic review	Women with breast cancer (n = 192)	MEDLINE, EMBASE, EMBASE Alert, BIOSIS, and PubMed from 1997-April 2018	5	Not specified	Not specified	Not used	Fatigue, hot flashes, sleep disturbance	Critically low	Recorded by some studies, but largely unrelated to the intervention
Lu et al., 2020 [49]	To conduct a meta-analysis on the effect of exercise on Aromatase Inhibitor Musculoskeletal Symtoms and quality of life in breast cancer survivors with AIs	Systematic review and meta-analysis	Women with breast cancer taking aromatase inhibitors (n = 665)	PubMed, Web of Science, EMBASE, CINAHL, Chinese Biomedical Service Platform(CBM), Wan Fang, Chinese Scientific and Technological Journal Database (VIP), and China National Knowledge Infrastructure (CNKI) from inception to May 1^st^ 2019	8	USA, China, UK, Spain	RCTs of quasi-experimental	Cochrane Risk of Bias Tool	Arthralgia	Moderate	No major adverse events, some joint and muscle pain with stiffness
Boing et al., 2020 [50]	To investigate the effects of exercise on physical outcomes in breast cancer women receiving any modality of hormone therapy	Systematic review and meta-analysis	Women with breast cancer using adjuvant hormonal therapy (n = 200)	PubMed, Web of Science, Cinahl Database, Cochrane Library for Clinical Trials and Lilacs	3	USA	Randomized or non-randomized	Cochrane Risk of Bias Tool and Risk of Bias in Non-Randomised Studies of Interventions	Arthralgia	Moderate	No adverse events reported
Li et al., 2016 [51]	To determine whether HM is effective and safe for reducing hot flushes and vasomotor symptoms, induced by endocrine therapy in patients with breast cancer	Systematic review and meta-analysis	Women with breast cancer using adjuvant hormonal therapy (n = 393)	MEDLINE, PubMed, EMBASE, PsycINFO, CINAHL, and the Cochrane Central Register of Control Trials, China National Knowledge Infrastructure (CNKI) and Wanfang from inception to July 2015	5	Venezuela and China	RCTs	Cochrane Risk of Bias Tool	Hot flashes, sleep disturbance	Moderate	4 minor adverse events in one study, no serious adverse events
Roberts et al., 2020 [59]	To assess the effects of exercise therapies on the prevention or management of aromatase inhibitor-induced musculoskeletal symptoms (AIMSS) in women with stage I to III hormone receptor-positive breast cancer	Systematic review and meta-analysis	Women with breast cancer using aromatase inhibitors (n = 353)	Cochrane Breast Cancer's Specialised Register, CENTRAL, MEDLINE, Embase and CINAHL, WHO, ICTRP, clinicaltrial.gov from inception to December 2018	6	USA, UK, Canada, Japan	RCTs	Cochrane Risk of Bias Tool	Arthralgia	High	No adverse events in 4 studies reporting them
Chan et al., 2020 [39]	The aim of the present review is to provide an overview on the findings of randomized control trials reporting the effectiveness of various types of interventions targeted for breast cancer survivors undergoing adjuvant endocrine therapy on the improvement of their QOL or health-related QOL (HRQOL) and functional ability	Systematic review	Women with breast cancer who have completed treatment other than adjuvant hormonal therapy (n = 1385)	PubMed, OVID MEDLINE (since 1946), EMBASE (since 1910), PsycINFO (since 1806), and CINAHL Complete from inception to November 2019	16	Brazil, France, Iran, Australia, Spain, USA, China	RCTs	Quality Assessment Tool for Quantitative Studies	Arthralgia, fatigue, hot flashes, sleep disturbance, vulvovaginal symptoms	Low	Not described
Liu et al., 2020 [52]	To undertake an updated systematic review and meta-analysis from all available RCTs to evaluate the effectiveness of tai chi for supportive care in patients with breast cancer	Systematic review and meta-analysis	Women with breast cancer who have received treatment (n = 207)	OVID MEDLINE, AMED, EMBASE, CINAHL, Cochrane Central Register of Controlled Trials, China National Knowledge Infrastructure (CNKI), VIP, and Wanfang Data from inception to June 2019	3	USA, Thailand	RCTs	Physiotherapy evidence databases scale	Fatigue, sleep disturbance	Moderate	Not described

*AIMSS* aromatase inhibitor-associated musculoskeletal syndrome; *CJP* Centuries Journal Project; *CMFD* China Masters’ Theses Full-text Database; *CPCD* China Proceedings of Conference Full-text Database; *CNKI* Chinese National Knowledge Infrastructure; *CEPS* Chinese Electronic Periodical Services; *ITPLS* Index to Taiwan Periodical Literature System; *WHO ICTRP* World Health Organisation International Clinical Trials Registry Platform; *QATSDD* Quality Assessment Tool for Studies with Diverse Designs; *EMBASE* Excerpta Medica Database; *CINAHL* Cumulative Index to Nursing and Allied Health Literature; *PEDro* Physiotherapy Evidence Database; *MEDLINE* Medical Literature Analysis and Retrieval System Online; *AMED* Allied and Complementary Medicine Database; *BNI* British Nursing Index; *CISCOM* Centralised Information Service for Complementary Medicine; *Hom-Inform* British Homeopathic Library; *SIGLE* System for Information on Grey Literature in Europe; *CENTRAL* Cochrane Central Register of Controlled Trials; *DOAJ* Directory of Open Access Journals; *EBSCO* EBSCO Information Services; *BMJ* British Medical Journal; *KSI* Korean Studies Information; *KISTI* Korea Institute of Science and Technology Information; *RICHB* Research Information Center for Health Database; *CPCD* China Proceedings of Conferences Database; *LILACS* Latin American and Caribbean Health Sciences Literature; *KSI* Korean Studies Information; Indexing of Indian Medical Journals (IndMED); International Congress on Complementary Medicine Research (ICCMR); *DAI* Dissertation Abstracts International

**Table 2:** Characteristics of clinical guidelines advising on self-management strategies for side-effects among women using AET

Organisation / date	Title	Country of origin	Intended audience	Reports methodology	Types of empirical evidence used	Reports evidence grading	Side-effect reported	Self-management strategies reported	Refers to peer-reviewed evidence
NCI, 2019 [62]	Hot Flashes and Night Sweats (PDQ®)–Health Professional Version	USA	Healthcare Professionals	Yes	Randomised trials, systematic reviews	Yes	Hot flashes and night sweats	Relaxation	Yes
								Behavioural strategies	No
								Vitamin E	Yes
								Soy	Yes
								Black Cohosh	Yes
								Flaxseed	Yes
								Magnesium oxide	Yes
								Acupuncture	Yes
ASCO, 2018 [63]	Integrative Therapies During and After Breast Cancer Treatment: ASCO Endorsement of the SIO Clinical Practice Guideline	USA	Healthcare professionals	Yes	Randomised trials	Yes	Fatigue^c^	Hypnosis	Yes^a^
								Ginseng	Yes^a^
								Acupuncture	Yes^a^
								Yoga	Yes^a^
								Acetyl-L-carnitine	Yes^a^
								Guarana	Yes^a^
							Hot flashes^c^	Acupuncture	Yes^a^
								Soy	Yes^a^
							Sleep disturbance^c^	Yoga	Yes^a^
CA, 2016 [71]	Management of menopausal symptoms in women with a history of breast cancer	Australia	Healthcare professionals and patients	Yes	Randomised trials	Yes	Hot flashes	Hypnosis	Yes
								Relaxation	Yes
								Physical activity	Yes
								Yoga	Yes
								Acupuncture	Yes
								Magnetic therapy	Yes
								Homeopathy	Yes
								Black cohosh	Yes
								Omega-3	Yes
							Sleep disturbance	Hypnosis	Yes
								Yoga	Yes
								Acupuncture	Yes
								Vitamin E	Yes
							Vulvovaginal symptoms	Non-hormonal lubricants and gels	Yes
NCI, 2016 [64]	Sleep disorders (PDQ)—Health professional version	USA	Healthcare Professionals	Yes	randomised trials	Yes	Sleep disturbance	Yoga	Yes
CA, 2016 [72]	Managing menopausal symptoms after breast cancer: a guide for women	Australia	Patients	No	Not reported	No	Arthralgia	Physical activity	No
								Vitamin supplements	No
							Fatigue	Physical activity	No
								Behavioural strategies	No
							Hot Flashes	Yoga	No
								Acupuncture	No
								Hypnotherapy	No
							Sleep Disturbance	Acupuncture	No
								Hypnotherapy	No
								Behavioural strategies	No
							Vulvovaginal Symptoms	Non-hormonal moisturisers, gels, lubricants	No
NAMS, 2015 [65]	Nonhormonal management of menopause-associated vasomotor symptoms: 2015 position statement of The North American Menopause Society^b^	USA	Healthcare Professionals	Yes	Observational studies, randomised trials, systematic reviews	Yes	Hot Flashes	Yoga	Yes
								Weight loss	Yes
								Hypnosis	Yes
AHS, 2015 [76]	Follow-up care for early stage breast cancer	Canada	Healthcare Professionals	Yes	Randomised trials, systematic reviews	No	Fatigue	Physical activity	No
								Behavioural strategies	No
							Vulvovaginal Symptoms	Non-hormonal moisturisers, lubricants	No
								Dilators	Yes
ACS/ASCO, 2016 [61]	American Cancer Society/American Society of Clinical Oncology Breast Cancer Survivorship Care Guideline	USA	Healthcare Professionals	Yes	Randomised trials, observational studies	Yes	Arthralgia	Acupuncture	Yes
								Physical activity	Yes
							Fatigue	Physical activity	Yes
							Hot Flashes	Behavioural strategies	No
							Vulvovaginal Symptoms	Non-hormonal lubricants and moisturisers	Yes
NICE, 2014 [74]	Tamoxifen—managing adverse effects	UK	Healthcare Professionals	Yes	Expert opinion	No	Hot Flashes	Behavioural strategies	No
							Sleep Disturbance	Behavioural strategies	No
							Nausea	Behavioural strategies	No
							Vulvovaginal Symptoms	Non-hormonal lubricant	No
NCI, 2017 [66]	Fatigue (PDQ®)–Health Professional Version	USA	Healthcare Professionals	Yes	Expert opinion, observational studies, randomised trials	Yes	Fatigue	Physical activity	Yes
								Behavioural strategies	No
ASCO, 2018 [67]	Interventions to Address Sexual Problems in People With Cancer: American Society of Clinical Oncology Clinical Practice Guideline Adaptation of Cancer Care Ontario Guideline	USA	Healthcare Professionals	Yes	Randomised trials	Yes	Vulvovaginal Symptoms	Physical activity	Yes
								Vaginal gel	Yes
								Dilators	Yes
ASCO, 2014 [68]	Screening, Assessment, and Management of Fatigue in Adult Survivors of Cancer: An American Society of Clinical Oncology Clinical Practice Guideline Adaptation	USA	Healthcare Professionals	Yes	Clinical guidelines, Society websites, systematic reviews	No	Fatigue	Physical activity	Yes
								Yoga	Yes
								Acupuncture	Yes
RCOG, 2010 [60]	Alternatives to HRT for the Management of Symptoms of the Menopause	UK	Healthcare Professionals	No	Randomised trials, systematic reviews	No	Hot flashes	Vitamin E	Yes
NCCN, 2020 [69]	NCCN Clinical Practice Guidelines in Oncology (NCCN Guidelines®) Cancer Related Fatigue	USA	Healthcare professionals	Yes	Randomised trials, clinical guidelines, systematic reviews	Yes	Fatigue	Physical activity	Yes
								Bright white light therapy	Yes
								Yoga	Yes
								Massage	Yes
CA, 2020 [73]	Guidance for the management of early breast cancer	Australia	Healthcare professionals	No	Not reported	No	Vulvovaginal symptoms	Vaginal lubricants	No
							Fatigue	Physical activity	No
							Arthralgia	Physical activity	No
							Sleep disturbance	Sleep hygiene practice	No
NAMS and ISSWSH, 2018 [70]	Management of genitourinary syndrome of menopause in women with or at high risk for breast cancer	USA	Healthcare professionals	Yes	Not reported	No	Vulvovaginal symptoms	Vaginal moisturisers	Yes
								Vaginal lubricants	Yes
								Vaginal dilators	Yes
ESMO, 2020 [77]	Cancer-related fatigue: ESMO clinical practice guidelines for diagnosis and treatment	Europe	Healthcare professionals	Yes	Not reported	Yes	Fatigue	Physical activity	Yes
								Yoga	Yes
								Acupuncture	Yes
								Guarana	Yes
								Mistletoe	Yes
BMS & ABS, 2018 [75]	The diagnosis of the menopause and management of oestrogen deficiency symptoms and arthralgia in women treated for breast cancer	UK	Healthcare professionals	No	Clinical guidelines, systematic reviews, and original research	No	Hot flashes	St John’s wort	Yes
								Behavioural strategies	Yes
								Soy and red clover	Yes
								Black cohosh	Yes
								Vitamin E	Yes
								Magnetic devices	Yes
							Vulvovaginal symptoms	Vaginal moisturisers	Yes
								Vaginal lubricants	Yes
							Arthralgia	Acupuncture	Yes
								Relaxation techniques	Yes
								Nutritional supplements	Yes

*ACS* American Cancer Society; *ACSM* American College of Sports Medicine; *AHS* Alberta Health Services; *ASCO* American Society of Clinical Oncology; *BMS* British Menopause Society; *CA* Cancer Australia; *ECPC* European Cancer Patient Coalition; *ES* Endocrine Society; *ESMO* European Society for Medical Oncology; *ISSWSH* International Society for the Study of Women’s Sexual Health; *IPOS* International Psycho-Oncology Society; *NAMS* North American Menopause Society; *NCCN* National Comprehensive Cancer Network; *NCI* National Cancer Institute; *NICE* National Institute for Health and Cancer Excellence; *RCOG* Royal College of Obstetrics & Gynaecologists), ^a^ = This guideline is an endorsement of the SIO Practice Guideline, which contains reference to peer-reviewed evidence; ^b^Only included evidence that had also been tested on women with breast cancer; ^c^This guideline also reported a large number of strategies that were described as having ‘insufficient evidence to form a clinical recommendation’ with no further discussion, citation or grading. They were therefore not discussed further in this review, but include: Acupressure, biofield healing, comprehensive coping strategy, CoQ10, Ganoderma lucidum, light treatment, massage, meditation, mind–body cognitive therapy, movement, multimodal, multivitamin, polarity therapy, stress management, qigong, reflexology, relaxation, stress management, yoga (Fatigue), Acupuncture, calendula cream, meditation, qigong, stress-management techniques (sleep disturbance), Black cohosh, flaxseed, homeopathy, hypnosis, magnetic therapy, meditation, peppermint, vitamin E, yoga (hot flashes)

### Evidence synthesis

Studies and guidelines were summarised in tables, and were classified into 3 categories: ‘X’ = Overall, no evidence or low-quality evidence in support of the intervention or high-quality evidence against the intervention; ‘?’ = mixed evidence with some evidence in favour of the intervention and some negative or null evidence against the intervention or no evidence provided for recommendation; **‘’** = Overall, more evidence or higher quality evidence in favour of the intervention than against it. We also undertook a narrative synthesis of the data. We first synthesized the data from the systematic reviews and guidelines independently, and then combined them to identify consistencies and discrepancies in terms of the strategies evaluated, and the conclusions drawn. There was high heterogeneity across the reviews and guidelines with regard to quality, strategies and side-effects included, and therefore no further quantitative synthesis was performed. We grouped reviews and guidelines thematically by side-effects, and self-management strategies reported. Higher quality reviews and guidelines were prioritised in the narrative synthesis when multiple data sources were available. However, lower quality reviews and guidelines were included to increase the sensitivity for identifying potential self-management strategies, and to enable us to provide recommendations for how progress can be made in this field.

The quality of systematic reviews was independently assessed using the AMSTAR-2 checklist by two authors (SS and LH), with disagreements resolved by a third author (KL). Seven domains assessed to produce a single quality score of critically low, low, moderate and high [25]. These include protocol registration, adequacy of the search, exclusion criteria justification, risk of bias from individual studies, appropriateness of meta-analytical methods, consideration of risk of bias when interpreting results, and assessment and impact of publication bias. Three authors (KL, SS and AB) independently used the AGREE-2 checklist to assess the quality of the clinical guidelines across six domains [26]. These include scope and purpose, stakeholder involvement, rigour of development, clarity of presentation, applicability, editorial independence. An average of their scores was computed. Guidelines with scores > 50% were considered ‘higher quality’.

### Changes from pre-registration

We did not extract data related to medication adherence, or broader outcomes related to side-effects such as quality of life or physical function. We also assessed the quality of the clinical guidelines using the AGREE-2 checklist. Some clinical guidelines also had separate document intended for patients rather than healthcare professionals. Where these included broadly identical content, we only included the healthcare professional versions.

## Results

### Description

The final database searches for systematic reviews retrieved 906 records. After duplicates were removed 580 titles and abstracts were screened and 70 full texts were reviewed. Thirty-three systematic reviews were eligible, including 10 identified via backward citation searching (Supplementary Figure S1). We identified 1426 clinical guidelines, and after deduplication 953 titles were screened, and 122 full guidelines were reviewed. Eighteen clinical guidelines were eligible, including one guideline identified via backwards citation searching (Supplementary Figure S2).

The quality of the systematic reviews was mixed. Four systematic reviews had critically low quality [27–30], nine were low quality [31–39], 13 moderate quality [40–52], and seven high quality [53–59] (Table 1). There were consistent weaknesses within the reviews. The quality assessment ratings for each systematic review are available in Appendix Table S1 and S2. No reviews reported the source of study funding, and pre-registrations and explanations for deviating from protocols was uncommon (six adherent [50, 54, 56–59], one partially adherent [40]). Six reviews failed to disclose author conflicts of interest or how they managed existing conflicts [29, 30, 32, 33, 46, 48]. The mean overall quality score of the clinical guidelines was 44%, ranging from 24 [60] to 71 [61] (Table 3). Guidelines scored particularly strongly on the ‘scope and purpose’ (mean = 72%) and ‘clarity of presentation’ (mean = 71%) domains, but ‘applicability’ (mean = 11%) and ‘editorial independence’ (24%) was lower.

**Table 3:** Overall and sub-scale average guideline quality scores as assessed by 3 reviewers completing the AGREE-2 assessment, %

	NCI Hot Flash [62]	ASCO integrative therapies [63]	Cancer Australia (Healthcare Professionals) [71]	NCI Sleep disorders [64]	Cancer Australia (Patients) [72]	NAMS [65]	AHS [76]	ACS and ASCO [61]	NICE [74]	NCI Fatigue [66]	ASCO sexual problems [67]	ASCO Fatigue [68]	RCOG [60]	NCCN [69]	Cancer Australia [73]	NAMS and ISSWSH [70]	ESMO Fatigue [77]	BMS and ABS [75]
Scope and purpose	61	81	81	59	78	57	87	87	74	61	96	98	43	56	80	78	35	80
Stakeholder involvement	31	65	72	31	46	39	30	78	63	33	80	80	13	39	43	43	22	43
Rigour and development	35	47	65	35	10	51	43	67	55	33	49	44	28	44	24	30	46	19
Clarity of presentation	46	78	83	46	76	85	70	83	67	50	96	81	52	70	89	72	69	70
Applicability	0	6	1	6	13	19	15	32	18	3	22	17	3	11	11	6	7	7
Editorial independence	22	54	40	18	0	37	41	49	20	13	27	19	0	0	14	38	33	0
Overall rating	32	52	58	33	32	49	47	71	52	33	63	56	24	40	40	45	40	33

Fifteen systematic reviews used a narrative synthesis [27, 29–31, 35–39, 42, 43, 45, 47, 53, 54], and 18 included at least one meta-analysis [28, 32–34, 40, 41, 44, 46, 48–52, 55–59]. On average, the articles included 7.9 (range of 2–26) relevant primary studies. The most common assessment of methodological quality was the Cochrane Risk of Bias Tool (*n* = 18) [31, 38, 40, 42–45, 47, 49–51, 53–59]. Reporting of adverse events resulting from the strategies evaluated was generally poor, and not reported by 11 systematic reviews [29, 34–37, 39, 47, 48, 52, 55, 58]. Reports from the remaining reviews indicated adverse events were minor (Table 1).

The majority of the guidelines originated in the USA (*n* = 10) [61–70], with the remainder originating in Australia (*n* = 3) [71–73], the UK (*n* = 3) [60, 74, 75], Canada (*n* = 1) [76], and Europe (*n* = 1) [77]. Sixteen guidelines were intended for healthcare professionals [60–70, 73–77], one for patients only [72], and one for both healthcare professionals and patients [71]. Fourteen guidelines explicitly reported their methodology for identifying their recommendations [61–71, 74, 76, 77]. When describing self-management strategies, seven referred to systematic reviews [60, 62, 65, 67, 68, 75, 76], 12 included randomised trials [60–67, 69, 71, 75, 76]; three referred to observational data [61, 65, 66] and two referred to expert opinion [66, 74]. One did not report the basis for the recommendation [72]. Ten provided a grading system for the strength of the evidence [61–67, 69, 71, 77].

### Arthralgia

Fourteen systematic reviews [27, 28, 31–33, 39–41, 44, 47–50, 59] and four clinical guidelines [61, 72, 73, 75] reported on five self-management strategies for arthralgia. One review was rated as high quality [59]. The guidelines scored between 32% [72] and 71% [61] in terms of quality. An overall summary of findings from the systematic reviews and clinical guidelines is shown in Table 4.4.

**Table 4:** Overall synthesis of findings from the systematic reviews and clinical guidelines broken down by symptom and self-management strategy

		**Systematic reviews**										
		Bae 2015 [31]	Chao 2009 [32]	Chen 2017 [40]	Chien 2015 [41]	Dowling 2017 [28]	Fritz 2013 [42]	Fritz 2014 [43]	Halsey 2015 [27]	Johns 2016 [53]	Kassab 2009 [54]	Nahm 2018 [47]
**Arthralgia**	Acupuncture	X	X	?	?	?			?			?
	Physical activity					?						?
	Supplements					X						?
	Relaxation											
	Miscellaneous											
**Gastrointestinal**	Acupuncture											
**Fatigue**	Acupuncture					?						
	Supplements							X				
	Physical activity					?						
	Behav strategies											
	Hypnosis											
	Miscellaneous											
**Hot flashes**	Acupuncture		X							?		
	Supplements						X	X		X	X	
	Hypnosis									X		
	Relaxation											
	Behav strategies											
	Physical activity											
	Magnetic therapy											
	Weight loss											
**Nausea**	Behav strategies											
**Vulvovaginal symptoms**	Gels, lubricants											
	Dilators											
	Physical activity											
	Supplements											
	Physical activity											
	Acupuncture											
	Hypnosis											
	Supplements											
	Behavioral strategies											
	Miscellaneous											
**Sleep**	Physical activity											
	Acupuncture											
	Hypnosis											
	Supplements											
	Behav strategies											
	Miscellaneous											

		**-Systematic reviews**										
		-Pan 2015 [55]	-Pan 2018 [44]	-Roberts 2017 [33]	-Salehi 2016 [34]	-Yang 2017 [48]	-Garcia 2015 [45]	-Lee 2009 [46]	-Rada 2010 [56]	-Tremblay 2008 [35]	-Cramer 2017 [57]	-Finnegan John 2013 [36]
**Arthralgia**	Acupuncture		?			✓						
	Physical activity	X		?		X						
	Supplements			X		X						
	Relaxation					?						
	Miscellaneous											
**Gastrointestinal**	Acupuncture		X									
**Fatigue**	Acupuncture		X									
	Supplements											?
	Physical activity										?	?
	Behavioral strategies											
	Hypnosis											
	Miscellaneous											
**Hot flashes**	Acupuncture		X		X		?	?	X	?		
	Supplements								X			
	Hypnosis											
	Relaxation								?	?		
	Behavioral strategies											
	Physical activity											
	Magnetic therapy								X			
	Weight loss											
**Nausea**	Behavioral strategies											
**Vulvovaginal symptoms**	Gels, lubricants											
	Dilators											
	Physical activity											
	Supplements											
	Physical activity											
	Acupuncture											
	Hypnosis											
	Supplements											
	Behavioral strategies											
	Miscellaneous											
**Sleep**	Physical activity											
	Acupuncture		X									✓
	Hypnosis											
	Supplements											
	Behavioral strategies											
	Miscellaneous											

		**-Systematic reviews**										
		-Taylor 2011 [37]	-Cramp 2012 [58]	-Bordeleau 2007 [29]	-Mazzarello 2015 [38]	-Ruan 2019 [30]	-Lu 2020 [49]	-Boing 2020 [50]	-Li 2016 [51]	-Roberts 2020 [59]	-Chan 2020 [39]	-Liu 2020 [52]
**Arthralgia**	Acupuncture										?	
	Physical activity						✓	?		?	?	
	Supplements										X	
	Relaxation											
	Miscellaneous										X	
**Gastrointestinal**	Acupuncture											
**Fatigue**	Acupuncture										?	
	Supplements					?					?	
	Physical activity		✓								?	?
	Behavioral strategies											
	Hypnosis											
	Miscellaneous										X	
**Hot flashes**	Acupuncture											
	Supplements			X		?			?		X	
	Hypnosis	?										
	Relaxation											
	Behav strategies											
	Physical activity											
	Magnetic therapy											
	Weight loss											
**Nausea**	Behavioral strategies											
**Vulvovaginal symptoms**	Gels, lubricants			✓								
	Dilators											
	Physical activity	X									X	
	Supplements										X	
	Physical activity											
	Acupuncture											
	Hypnosis	?										
	Supplements											
	Behavioral strategies											
	Miscellaneous											
**Sleep**	Physical activity											X
	Acupuncture					?						
	Hypnosis											
	Supplements								?			
	Behavioral strategies											
	Miscellaneous											

		**-Clinical guidelines**										
		-NCI, 2019 [62]	-ASCO, 2018 [63]	-CA, 2016 [71]	-NCI, 2016 [64]	-CA, 2016 [72]	-NAMS, 2015 [65]	-AHS, 2015 [76]	-ACS/ASCO, 2016 [61]	-NICE, 2014 [74]	-NCI, 2017 [66]	-ASCO, 2018 [67]
**Arthralgia**	Acupuncture								?			
	Physical activity					?			?			
	Supplements					?						
	Relaxation											
	Miscellaneous											
**Gastrointestinal**	Acupuncture											
**Fatigue**	Acupuncture		?									
	Supplements		X									
	Physical activity		?			?		?	✓		✓	
	Behavioral strategies					?		?			?	
	Hypnosis		?									
	Miscellaneous											
**Hot flashes**	Acupuncture	?	?	?		?						
	Supplements	?	X	X								
	Hypnosis			?		?	?					
	Relaxation	?		?								
	Behavioral strategies	?							?	?		
	Physical activity			?		?	X					
	Magnetic therapy			X								
	Weight loss						?					
**Nausea**	Behavioral strategies									?		
**Vulvovaginal symptoms**	Gels, lubricants			✓		?		✓	✓	✓		✓
	Dilators							✓				✓
	Physical activity											✓
	Supplements											
	Physical activity											
	Acupuncture											
	Hypnosis											
	Supplements											
	Behavioral strategies											
	Miscellaneous											
**Sleep**	Physical activity		?	?	?					?		
	Acupuncture			?		?						
	Hypnosis			?		?						
	Supplements			X								
	Behavioral strategies					?						
	Miscellaneous											

		**-Clinical guidelines**						
		-ASCO, 2014 [68]	-RCOG, 2010 [60]	-NCCN, 2020 [69]	-CA, 2020 [73]	-NAMS & ISSWSH, 2018 [70]	-ESMO, 2020 [77]	-BMS & ABS, 2018 [75]
**Arthralgia**	Acupuncture							✓
	Physical activity				?			
	Supplements							X
	Relaxation							✓
	Miscellaneous							
**Gastrointestinal**	Acupuncture							
**Fatigue**	Acupuncture	?					?	
	Supplements						X	
	Physical activity	?		✓	?		✓	
	Behavioral strategies							
	Hypnosis							
	Miscellaneous			?				
**Hot flashes**	Acupuncture							
	Supplements		?					X
	Hypnosis							
	Relaxation							
	Behavioral strategies							?
	Physical activity							
	Magnetic therapy							X
	Weight loss							
**Nausea**	Behavioral strategies							
**Vulvovaginal symptoms**	Gels, lubricants				?	✓		✓
	Dilators					✓		
	Physical activity							
	Supplements							
	Physical activity							
	Acupuncture							
	Hypnosis							
	Supplements							
	Behavioral strategies							
	Miscellaneous							
**Sleep**	Physical activity							
	Acupuncture							
	Hypnosis							
	Supplements							
	Behavioral strategies				?			
	Miscellaneous							

X = Overall, no evidence or low-quality evidence in support of the intervention or high-quality evidence against the intervention; ? = Overall mixed evidence with some evidence in favour of the intervention and some negative or null evidence against the intervention or no evidence provided for recommendation; ✓ = Overall, more evidence or higher quality evidence in favour of the intervention than against it.

#### Acupuncture

Eleven systematic reviews reported on the use of acupuncture for arthralgia [27, 28, 31–33, 39–41, 44, 47, 48]. There were no high-quality reviews. Two moderate quality reviews [40, 44], one higher quality clinical guideline [61], and one lower quality clinical guideline [75] reported evidence supporting the use of acupuncture for arthralgia, but multiple limitations were noted with studies in this area. These included a tendency to report within group differences rather than between group differences in trial outcomes, short-term follow-up and small sample sizes. A third moderate quality review and meta-analysis reported the effect of acupuncture on arthralgia was not statistically significant in the five trials synthesised.

#### Physical activity

Eight systematic reviews [28, 33, 39, 47–50, 59] and three clinical guidelines [61, 72, 73] reported on physical activity as a self-management strategy for arthralgia. One systematic review [59] and one clinical guideline [61] were rated as high quality. Activities reported included yoga [28, 33, 39, 47, 48, 72], walking [28, 33, 47–49, 59, 72], Nordic walking [33, 48], aerobic and resistance training [28, 33, 39, 47–50, 59, 61, 73], aquatic exercise [33, 49, 72], and Tai-Chi [33]. A high-quality review reported no clear evidence of benefit for exercise therapy to address arthralgia [59]. This finding was supported by most evidence from the lower quality reviews, particularly those focussed on low intensity interventions such as yoga and Tai-Chi. One large randomised controlled trial was included in all systematic reviews [28, 33, 39, 47–50, 59] and one of the clinical guidelines [72], and reported supervised aerobic and resistance training could improve arthralgia in this population.

#### Supplements

Two moderate quality systematic reviews [47, 48], two low-quality systematic reviews [33, 39] and a critically low-quality systematic review [28] reported on the use of supplements to address arthralgia. Two lower quality clinical guidelines also reported on the use of supplements for this indication [75], of which one was patient-facing and did not refer to any evidence [72]. Supplements reported included vitamin D [28, 33], vitamin E [33], omega-3 fatty acids [33, 39, 47, 75], Glucosmine sulfate [33, 47, 75], chondroitin-sulfate [47], kampo medicine [47], sodium selenite plant enzmes [47], lens culinaris lectin [47], Blue Citrus Herbal [33], high dose vitamin B2 [75], and unspecified vitamins [72]. The weight of evidence suggested supplements are unlikely to be effective at improving arthralgia (Table 4). The majority of primary data referred to within the reviews and guidelines were from weak study designs and small sample sizes. Observational designs tended to yield larger effects in favour of supplement use.

#### Relaxation

One lower quality clinical guideline reported evidence from a systematic review and meta-analysis suggesting a moderate to large effect of relaxation techniques on arthralgia [75]. However, no details were provided on techniques or quality of the evidence.

#### Miscellaneous

One low-quality systematic review identified a single trial of whole body vibration, which indicated no effect on a range of arthralgia outcomes [39].

### Fatigue

Eight systematic reviews [28, 36, 39, 43, 44, 55, 57, 58] and nine clinical guidelines [61, 63, 66, 68, 69, 72, 73, 76, 77] referred to self-management strategies for fatigue. Three systematic reviews were rated as high quality [55, 57, 58]. Three clinical guidelines scored > 50% with regard to quality [61, 63, 68].

#### Acupuncture

Three systematic reviews [36, 39, 44], two higher quality clinical guidelines [63, 68], and one lower quality clinical guideline [77] reported on the use of acupuncture to support fatigue. There were no high-quality reviews. In a moderate quality systematic review and meta-analysis of four studies, acupuncture was reported to be ineffective at managing fatigue in women with breast cancer [44]. One guideline graded acupuncture as ‘C’ whereby ‘clinicians are advised to selectively recommend offering acupuncture to patients based on professional judgement and patient preference [63].’ A second guideline reported evidence from two randomised trials of acupuncture, indicating it may relieve fatigue, but without an evidence grading [68]. A lower quality guideline reported evidence supporting the use of acupuncture for fatigue, but the guideline panel could not reach a consensus in its recommendation [77].

#### Supplements

Three systematic reviews [30, 39, 42] and two clinical guidelines [63, 77] reviewed at least one supplement as a strategy for managing fatigue. A moderate quality systematic review [43] and a critically low-quality systematic review [30] investigated the use of black cohosh to address cancer-related fatigue. The moderate quality review identified a single observational study, which demonstrated no association between the supplement and fatigue [43]. The low-quality systematic review identified a single randomised controlled trial at high risk of bias, testing Chinese herbs for fatigue [36]. While changes to fatigue were observed in the intervention group, the review concluded this effect should be interpreted with caution. A low-quality systematic review reported data from a single trial indicating spore powder of G. Lucidum improved fatigue in this population [39]. A clinical guideline graded the evidence for Ginseng as ‘Grade C’ indicating equivocal evidence. The same guideline also discouraged the use of Acetyl-L-carntine and Guarana due to moderate or high certainty that there is no net benefit (Grade D) [63]. A lower quality guideline did not recommend guarana or mistletoe extract for fatigue [77].

#### Physical activity

Seven systematic reviews [28, 36, 39, 52, 55, 57, 58] and nine clinical guidelines [61, 63, 66, 68, 69, 72, 73, 76, 77] included physical activity interventions for fatigue. One high-quality systematic review investigated the effect of Tai Chi exercise on physical wellbeing, mostly measured using the Chronic Illness Therapy – Fatigue survey [55]. In their meta-analysis of five trials, no overall beneficial effect was observed. This finding is supported by a second high-quality systematic review [58]. A third high-quality review concluded yoga could reduce fatigue in the short term but not medium-term compared with no therapy [57]. It also reported there was very low-quality evidence to suggest yoga is comparable to other physical activity interventions. A low-quality review found no evidence for effects of other low intensity physical activity interventions, such as massage therapy [36]. Four clinical guidelines recommended yoga [63, 68, 69, 77] (AGREE-2 range = 40–56%).

A high-quality review demonstrated that aerobic exercise was consistently beneficial for fatigue, but there was weak and inconclusive effects for resistance training [58]. One lower quality clinical guideline recommended combining aerobic and resistance exercise (category 1. High-level evidence and consensus that it was appropriate) [69]. Five guidelines recommended unspecified physical activity [61, 66, 68, 69, 72, 73, 76] (AGREE-2 range = 32–71%), although three provided no evidence for this recommendation [72, 73, 76]. In two mixed quality clinical guidelines, the evidence provided was graded as level I (recommendation based on meta-analysis) [61] and level II (recommendation based on non-randomised trials or observational data) [66].

#### Behavioural strategies

Three lower quality clinical guidelines recommended a range of strategies, including avoiding long periods in bed [72], consuming enough fruit, vegetables and water [72], and rest [66, 76]. None were supported by peer-reviewed evidence.

#### Hypnosis

One clinical guideline included hypnosis as a strategy for managing fatigue [63] (AGREE-2 score = 52%). Its grading (grade C) indicated clinicians should selectively offer hypnosis to patients. No systematic reviews were identified for hypnosis and fatigue management.

#### Miscellaneous

A low-quality systematic review reported a single study of neuromuscular taping which demonstrated s lower fatigue in the intervention group compared with a control group at 5-weeks post-intervention [39]. The same review also reported data from a single trial reporting no effect of whole body vibration on fatigue.

### Hot flashes

Hot flashes were the most common symptom covered by systematic reviews (*n* = 13) [29, 32, 34, 35, 37, 42–46, 53, 54, 56] and clinical guidelines (*n* = 9) [60–63, 65, 71, 72, 74, 75]. Three systematic reviews were rated as high quality [53, 54, 56]. Four clinical guidelines were higher quality [61, 63, 71, 74].

#### Acupuncture

Eight systematic reviews [32, 34, 35, 44–46, 53, 56] and four clinical guidelines [62, 63, 71, 72] reported on the use of acupuncture for managing hot flashes. Three systematic reviews were rated as low quality [32, 34, 35], three as moderate quality [44–46] and two as high quality [53, 56]. The quality of the guidelines ranged from 32 to 58%. The high-quality reviews concluded there was either no evidence for acupuncture for this indication [56] or there was no effect of acupuncture when compared with active pharmacological interventions [53]. Within-group analysis and a lack of long-term follow-up were consistent limitations within trials. The clinical guidelines indicated mixed evidence for acupuncture for managing hot flashes [62, 63, 71, 72] (Table 4).

#### Supplements

Nine systematic reviews [29, 30, 39, 42, 43, 51, 53, 54, 56] and five clinical guidelines [60, 62, 63, 71, 75] reported 10 different supplements for the self-management of hot flashes. Supplements included vitamin E [29, 53, 56, 60, 62], soy [29, 42, 62, 63, 75], black cohosh [29, 30, 43, 62, 71], Omega 3 [71], Flaxseed [62], magnesium oxide [62], homeopathy [39, 54, 56, 71], herbal medicine [51], medicinal plant extract (guarana) [39], and St John’s wort [30, 75]. The overall weight of evidence within the systematic reviews indicated no effect of supplements on hot flashes, with widespread study weaknesses within the field (Table 4).

#### Hypnosis

Hypnosis was investigated as a strategy to support hot flashes in two systematic reviews [37, 53], and was also included in three clinical guidelines [65, 71, 72]. A high-quality systematic review identified only one relevant trial that was stopped prematurely due to recruitment difficulties [53]. One higher quality clinical guideline reported the body of evidence was weak and cautiously suggested hypnotherapy could be considered (grade D) [71], and a second lower quality guideline cited the same clinical trial suggesting hypnosis could be a promising strategy for managing hot flashes. They graded the evidence as ‘Level 1’ (i.e. based on high-quality trials or systematic reviews) [65].

#### Relaxation

Two systematic reviews [35, 56] and two clinical guidelines [62, 71] included relaxation as a strategy for self-managing hot flashes. The systematic reviews were rated as high [56] and low [35] quality. The clinical guidelines scored 32% [62] and 58% [71] using the AGREE-2 checklist. Both reviews included two relevant studies, reporting mixed evidence for effects on hot flashes. There was a tendency to report within-group rather than between-group differences within the individual trials. One guideline reported absent effects in trials with adequate control arms [62], while the other indicated limited evidence of reductions in hot flash frequency and severity [71].

#### Behavioural strategies

Four clinical guideline recommended a range of strategies, including lifestyle and environment modifications [61, 62, 74, 75]. Suggestions included avoiding caffeine, alcohol and spicy foods, sipping cool drinks, reducing stress, changing to a cool room temperature, use of portable fans, opening windows, weight loss, wearing loose cotton clothing, and dressing in layers. No evidence was provided for these recommendations.

#### Physical activity

A moderate quality systematic review concluded Tai Chi was ineffective for hot flashes [52]. Three clinical guidelines reported on physical activity for hot flashes, all focusing on yoga [65, 71, 72] (AGREE-2 range = 32–58%). One guideline indicated no effect of yoga on vasomotor symptoms [65], while a second recommended it without referring to peer-reviewed evidence [72]. The final guideline reported two individual trials among women with breast cancer indicating yoga may reduce the frequency and severity of hot flushes and, when combined with meditation, also improve overall menopausal symptoms [71]. Both trials were assessed by the guideline developers as having a moderate risk of bias.

#### Magnetic therapy

No systematic reviews and two clinical guidelines reported on the use of magnetic therapy for the treatment of hot flashes [71, 75] (AGREE-2 scores = 58% and 33%). One clinical guideline reported a single randomised controlled trial indicating no effect of magnetic therapy, with the placebo arm reporting a lower frequency of hot flashes at follow-up. This was graded as a ‘C’ quality of evidence, indicating cautious application of the recommendation. The second clinical guideline concluded magnetic devices are not advised for this population.

#### Weight loss

No systematic reviews were identified reporting on the role of weight loss and hot flashes, and one clinical guideline did report on this [65] (AGREE-2 score = 49%). The authors cited a secondary analysis of a randomised dietary trial, indicating women with breast cancer who had lost at least 10% of their bodyweight during the 2-year follow-up were less likely to report moderate or severe vasomotor symptoms. The guideline indicated the evidence for weight loss on vasomotor symptoms (not distinguishing breast cancer and general population samples) was level II, indicating it may be beneficial for alleviating vasomotor symptoms.

### Vulvovaginal symptoms

Three low-quality systematic reviews were identified for vulvovaginal symptoms [37–39], and self-management strategies to address these symptoms were included in nine clinical guidelines [61, 67, 70–76]. Four guidelines scored > 50% with regard to quality [61, 67, 71, 74].

#### Moisturisers, gels, and lubricants

A low-quality systematic review [38] and nine clinical guidelines of mixed quality [61, 67, 70–76] all supported the use of moisturisers, gels or lubricants for this indication. Three guidelines did not refer to peer-reviewed literature [72–74]. The systematic review reported reductions in vaginal dryness and dyspareunia when using a pH-balanced gel and a vaginal moisturiser [38], and reduced dyspareunia when using aqueous lidocaine. A higher quality clinical guideline recommended the use of lubricants for all sexual touch in addition to frequent application of vaginal moisturiser [67]. A second higher quality clinical guideline reported a 1A level of evidence (based on RCTs) for non-hormonal water-based lubricants and moisturisers for vaginal dryness [61].

#### Vaginal dilators

Three clinical guidelines recommended the use of dilators to address vaginismus and vaginal stenosis [67, 70, 76]. It was not clear whether these recommendations were informed by primary research undertaken with women affected by breast cancer.

#### Physical activity

A systematic review rated as low quality [37] and a higher quality clinical guideline [67] reported on the evidence for physical activity and vulvovaginal symptoms. The systematic review reported two randomised trials of exercise interventions that had no effect on sexual functioning. The clinical guideline focussed on general exercise and pelvic floor exercises and reported these may be effective to support vulvovaginal discomfort and lower urinary tract symptoms. It was unclear what primary research informed this recommendation.

#### Supplements

A low-quality systematic review reported there was no effect of medicinal plant extract (guarana) on sexual function [39].

### Sleep disturbance

Six systematic reviews of varying quality [30, 39, 44, 51, 52, 57] and five clinical guidelines [63, 71–74] reported on strategies to address sleep disturbance in women with breast cancer. Three guidelines scored > 50% with regard to quality [63, 71, 74].

### Physical activity

A high-quality systematic review identified eight relevant studies comparing yoga to no therapy (*n* = 6) or psychological interventions (*n* = 2) [57]. It concluded yoga is favourable in comparison with no therapy, but not psychological interventions for sleep disturbance. A moderate-quality systematic review also indicated there was no long-term effect of Tai Chi on sleep [52]. A low-quality systematic review reported physical activity interventions may improve several sleep-related outcomes, although the interventions were poorly defined [39]. A clinical guideline reported there was equivocal evidence or small net benefit (grade C) for gentle yoga in improving sleep quality [63] (AGREE-2 score = 52%).

#### Acupuncture

A moderate quality systematic review reported a single acupuncture trial demonstrating this intervention did not significantly improve sleep quality [44]. Two clinical guidelines included acupuncture for sleep disturbance [71, 72], although only one referred to peer-reviewed evidence [71]. This guideline cited a single randomised trial, which indicated acupuncture improved sleep quality compared with sham acupuncture, but only within group differences were reported.

#### Hypnosis

Two clinical guidelines included hypnosis for sleep disturbance, although only one referred to peer-reviewed evidence [71, 72]. This reported a single randomised trial with a moderate risk of bias indicating hypnotherapy improved sleep quality when compared with a no treatment control [71].

#### Supplements

A low-quality systematic review reported herbal medicine may alleviate insomnia [51]. A systematic review rated as critically low quality reported black cohosh (with or without St John’s wort) may be effective at improving sleep disturbance. A higher quality clinical guideline reported a single randomised trial comparing gabapentin with vitamin E over 12 weeks [71]. Their findings indicated vitamin E had no effect on sleep disturbance compared with gabapentin.

#### Behavioural strategies

Three clinical guidelines recommended a range of behavioural strategies to improve sleep quality, although none were supported by peer-reviewed evidence [72–74]. These included sleep hygiene techniques, such as not exercising late in the day, maintaining a regular bedtime, avoiding stimulants (e.g. caffeine, alcohol), restricting the bedroom for sleep, avoiding napping, and keeping the bedroom cool.

#### Miscellaneous

A low-quality systematic review reported neuromuscular taping did not reduce sleep disturbance [39].

### Gastrointestinal discomfort

One high-quality systematic review and meta-analysis reported on the use of acupuncture to manage gastrointestinal discomfort [44]. No clinical guidelines were identified.

#### Acupuncture

Five studies were included in the meta-analysis, which showed no benefit of acupuncture on gastrointestinal discomfort [44].

### Nausea

One clinical guideline reported on the topic of nausea induced by AET [74] (AGREE-2 score = 52%). No systematic reviews were identified.

#### Behavioural strategies

The NICE clinical guideline recommended that women should take tamoxifen with food or milk, or at night [74]. No empirical evidence was provided.

## Discussion

In this umbrella review of clinical guidelines and systematic reviews, there was no clear evidence to support the majority of self-management strategies currently recommended and used by breast cancer survivors experiencing side-effects from AET. Unanimous agreement was only observed for moisturisers, lubricants and gels for women affected by vulvovaginal symptoms, where a systematic review and seven clinical guidelines endorsed this strategy. Considering the prevalence of side-effects of AET [2–6], and that over a quarter of women with these symptoms attempt self-management, there should be a renewed focus in evaluating and implementing support for breast cancer survivors [6]. Healthcare professionals supporting the management of breast cancer survivors should counsel women that the evidence supporting most self-management strategies for AET side-effects have a weak and inconsistent evidence base. While many strategies are low risk, patients should be encouraged to seek healthcare professional support if they do not alleviate their symptoms, as evidence supporting pharmacological interventions appears stronger [78].

Despite the wide-ranging benefits of physical activity observed among cancer populations [79–81], and its inclusion in multiple clinical guidelines identified here [61, 63, 65, 66, 68, 69, 71–73, 76, 77], there was often weak or inconclusive evidence from systematic reviews to support this strategy for arthralgia, fatigue, hot flashes, vulvovaginal symptoms and sleep disturbance in women affected by breast cancer using AET. This was particularly the case for low intensity activities such as Tai Chi and yoga. Among the high-quality systematic reviews, yoga [57] and aerobic exercise [58] was shown to be effective for fatigue, although yoga may only be beneficial in the short-term. The conclusion that the evidence for physical activity for self-managing AET symptoms is weak or inconclusive was made on the basis that the majority of the primary research informing these reviews was affected by either low statistical power, poor study design or inadequate follow-up. A rare example is a trial led by Irwin and colleagues [82] which strongly influenced the evidence to support physical activity for arthralgia within the reviews and guidelines. Such trials suggest it would be premature to conclude that physical activity is ineffective for self-managing AET, but similar high-quality studies are needed. Specifically, further information is needed to inform precise recommendations for physical activity type, duration and intensity of physical activity. Reporting guidelines such as the Consensus on Exercise Reporting Template (CERT) could be also more widely used to improve the specificity of reporting [83].

We aimed to observe the extent to which guidelines adequately reflected scientific evidence generated in systematic reviews. Strategies with at least some supporting evidence for a beneficial effect were generally appropriately reflected in the clinical guidelines, as indicated by moisturisers, gels and lubricants, and physical activity. However, there were examples of clinical guidelines introducing recommended strategies not supported by systematic review evidence. For example, weight loss and physical activity for hot flashes, hypnosis for fatigue and sleep, and behavioural strategies for fatigue, hot flashes, and sleep. In the case of dilators for vulvovaginal symptoms, the clinical guidelines largely relied on narrative reviews and early phase pilot trials, and our search did not identify any systematic reviews in this area. A systematic review of dilators for this indication could therefore be warranted.

The reviews and guidelines identified observed consistent weaknesses with the primary research they included, which often led us to conclude that the evidence for strategies was inconclusive. The use of acupuncture for arthralgia is a useful example to illustrate this point. Weak study designs were common, including observational studies with a high risk of bias and underpowered pilot trials that relied upon within-group rather than between-group comparisons. Short-term follow-up within clinical trials was also widespread, which can limit the extent to which these data apply to women using AET over a 5- to 10-year period [84]. These weaknesses were common, but not ubiquitous, and the mixed evidence may explain why others have concluded this strategy to be efficacious for this indication [78]. Adequately powered trials of promising supportive care interventions with long-term follow-up are needed to inform clinical guidelines.

There were also weaknesses with regard to the reviews that we identified. Overall, 13 (39%) systematic reviews were rated as either low or critically low quality. Our finding that duplicate data extraction was only present in less than half of the reviews is particularly problematic. There was a high degree of overlap with regard to the strategies assessed for specific indications within the systematic reviews. For example, we identified 11 systematic reviews reporting on the use of acupuncture for arthralgia. This is a form of research waste, as evidenced by so few clinical guidelines including acupuncture for arthralgia management. This duplication could be addressed through pre-registration of systematic review protocols.

The quality of some clinical guidelines was also low, and they often made recommendations without citing evidence or reporting evidence for strategies that had only been tested in healthy populations or different clinical groups. Most guidelines failed to consider potential barriers to implementation within routine clinical care. Guideline developers should consider describing the resource implications of applying recommendations and suggest audit criteria to monitor and drive implementation. Furthermore, editorial independence was assessed as being particularly poor for the guidelines identified. To improve transparency within guideline development, the views of the funding body and competing interests of the contributors should be disclosed and easy to identify.

Our decision to include both systematic reviews and clinical guidelines enabled us to produce a more comprehensive review with a broader range of self-management strategies. However, a limitation that should be acknowledged is that synthesizing the data from each document type simultaneously was often challenging because the purpose of each document is inherently different. For example, the clinical guidelines endorsing physical activity were likely to consider the wide-ranging benefits observed among cancer populations [79–81], and therefore their recommendations may not have been based solely on the evidence for self-managing symptoms of AET. This wider context should be considered when comparing guidelines with the findings of systematic reviews. Our ability to compare each document type was further limited by the differences between the quality assessment tool we used for each, making the quality scores difficult to compare. We included all reviews and guidelines regardless of the quality scores they were given. While we gave greater weight to the higher quality reviews and guidelines within our narrative synthesis, different conclusions may have been drawn had we excluded publications considered to be lower quality.

Further limitations are that we excluded pharmacological approaches and strategies that would require healthcare professional supervision, many of which have known efficacy [78]. Patients should be encouraged to seek healthcare professional support if the initial self-management strategies are ineffective. For pragmatic reasons we did not search all databases for the clinical guidelines, and this may also have led us to miss important documents. Side-effects such as nausea and fatigue are associated with a range of breast cancer treatments, but our review focussed on literature involving women using AET. Effective self-management interventions tested among women experiencing these symptoms who are not using AET could also be useful, but may require evaluation before being widely recommended. Finally, a limitation of the umbrella review method overall is that too much weight is placed on primary studies that are cited across multiple reviews and guidelines. This should be considered when interpreting our findings.

In conclusion, we identified a large body of systematic reviews and clinical guidelines of self-management strategies for women experiencing side-effects from AET. However, unanimous agreement for a strategy was only observed for moisturisers, gels and lubricants for women experiencing vulvovaginal symptoms. Evidence supporting physical activity for AET symptoms was only clear for aerobic activity and yoga for alleviating fatigue. Breast cancer survivors should therefore be cautious about using most self-management strategies currently recommended for AET side-effects. Healthcare professionals should be aware that although the risk of harm is unlikely with most of these strategies, the likelihood of benefit is often unclear. Primary research in this area relied heavily on inadequately powered trials using poor study designs. A large proportion of the systematic reviews and clinical guidelines identified were also of lower quality. There is a need for high-quality longitudinal primary research of promising self-management interventions for women with breast cancer using AET. Findings from such trials should be incorporated within clinical guidelines that have considered how to implement these strategies into routine practice.

## Supplementary Information

Below is the link to the electronic supplementary material.

Supplementary file1 (DOCX 36 kb)

Supplementary file2 (DOCX 56 kb)

Supplementary file3 (DOCX 33 kb)

## Data Availability

Template data collection forms, data extracted from included studies, and any other materials used in the review are available from the corresponding author upon reasonable request.

## References

[1] Bray F, Ferlay J, Soerjomataram I, Siegel RL, Torre LA, Jemal A (2018). Global cancer statistics 2018: GLOBOCAN estimates of incidence and mortality worldwide for 36 cancers in 185 countries. CA Cancer J Clin.

[2] Cella D, Fallowfield LJ (2008). Recognition and management of treatment-related side effects for breast cancer patients receiving adjuvant endocrine therapy. Breast Cancer Res Treat.

[3] Howell A, Cuzick J, Baum M, Buzdar A, Dowsett M, Forbes JF (2005). Results of the ATAC (Arimidex, Tamoxifen, Alone or in Combination) trial after completion of 5 years’ adjuvant treatment for breast cancer. Lancet.

[4] Jakesz R, Jonat W, Gnant M, Mittlboeck M, Greil R, Tausch C (2005). Switching of postmenopausal women with endocrine-responsive early breast cancer to anastrozole after 2 years’ adjuvant tamoxifen: combined results of ABCSG trial 8 and ARNO 95 trial. Lancet.

[5] Breast International Group (BIG) 1–98 Collaborative Group, Thürlimann B, Keshaviah A, Coates AS, Mouridsen H, Mauriac L, et al. A comparison of letrozole and tamoxifen in postmenopausal women with early breast cancer. N Engl J Med 2005; 353: 2747–57. 10.1056/NEJMoa052258. 10.1056/NEJMoa052258 16382061

[6] Peate M, Saunders C, Cohen P, Hickey M (2021). Who is managing menopausal symptoms, sexual problems, mood and sleep disturbance after breast cancer and is it working? Findings from a large community-based survey of breast cancer survivors. Breast Cancer Res Treat.

[7] Fallowfield L, Cella D, Cuzick J, Francis S, Locker G, Howell A (2004). Quality of life of postmenopausal women in the Arimidex, Tamoxifen, Alone or in Combination (ATAC) adjuvant breast cancer trial. J Clin Oncol.

[8] Ganz PA, Petersen L, Bower JE, Crespi CM (2016). Impact of adjuvant endocrine therapy on quality of life and symptoms: observational data over 12 Months from the mind-body study. J Clin Oncol.

[9] Schover LR, Baum GP, Fuson LA, Brewster A, Melhem-Bertrandt A (2014). Sexual problems during the first 2 years of adjuvant treatment with aromatase inhibitors. J Sex Med.

[10] Demissie S, Silliman RA, Lash TL (2001). Adjuvant tamoxifen: predictors of use, side effects, and discontinuation in older women. J Clin Oncol.

[11] Kahn KL, Schneider EC, Malin JL, Adams JL, Epstein AM (2007). Patient centered experiences in breast cancer: predicting long-term adherence to tamoxifen use. Med Care.

[12] Murphy CC, Bartholomew LK, Carpentier MY, Bluethmann SM, Vernon SW (2012). Adherence to adjuvant hormonal therapy among breast cancer survivors in clinical practice: a systematic review. Breast Cancer Res Treat.

[13] Brett J, Boulton M, Fenlon D, Hulbert-Williams NJ, Walter FM, Donnelly P (2018). Adjuvant endocrine therapy after breast cancer: a qualitative study of factors associated with adherence. Patient Prefer Adherence.

[14] Wheeler SB, Roberts MC, Bloom D, Reeder-Hayes KE, Espada M, Peppercorn J (2016). Oncology providers’ perspectives on endocrine therapy prescribing and management. Patient Prefer Adherence.

[15] Winn AN, Dusetzina SB (2016). The association between trajectories of endocrine therapy adherence and mortality among women with breast cancer. Pharmacoepidemiol Drug Saf.

[16] McCowan C, Wang S, Thompson AM, Makubate B, Petrie DJ (2013). The value of high adherence to tamoxifen in women with breast cancer: a community-based cohort study. Br J Cancer.

[17] Makubate B, Donnan PT, Dewar JA, Thompson AM, McCowan C (2013). Cohort study of adherence to adjuvant endocrine therapy, breast cancer recurrence and mortality. Br J Cancer.

[18] Hershman DL, Kushi LH, Shao T, Buono D, Kershenbaum A, Tsai WY (2010). Early discontinuation and nonadherence to adjuvant hormonal therapy in a cohort of 8,769 early-stage breast cancer patients. J Clin Oncol.

[19] Yood MU, Owusu C, Buist DSM, Geiger AM, Field TS, Thwin SS (2008). Mortality impact of less-than-standard therapy in older breast cancer patients. J Am Coll Surg.

[20] Early Breast Cancer Trialists’ Collaborative Group (EBCTCG), Davies C, Godwin J, Gray R, Clarke M, Cutter D, et al. Relevance of breast cancer hormone receptors and other factors to the efficacy of adjuvant tamoxifen: patient-level meta-analysis of randomised trials. Lancet 2011;378:771–84. 10.1016/S0140-6736(11)60993-8. 10.1016/S0140-6736(11)60993-8 PMC3163848 21802721

[21] Haque R, Ahmed SA, Fisher A, Avila CC, Shi J, Guo A (2012). Effectiveness of aromatase inhibitors and tamoxifen in reducing subsequent breast cancer. Cancer Med.

[22] Aromataris E, Fernandez R, Godfrey C, Holly C, Khalil H, Tungpunkom P. Chapter 10: Umbrella Reviews. JBI Man. Evid. Synth., 2020. 10.1097/XEB.0000000000000055 26360830

[23] Page MJ, McKenzie JE, Bossuyt PM, Boutron I, Hoffmann TC, Mulrow CD (2021). The PRISMA 2020 statement: an updated guideline for reporting systematic reviews. BMJ.

[24] McGowan J, Sampson M, Salzwedel DM, Cogo E, Foerster V, Lefebvre C (2016). PRESS Peer Review of Electronic Search Strategies: 2015 Guideline Statement. J Clin Epidemiol.

[25] Shea BJ, Reeves BC, Wells G, Thuku M, Hamel C, Moran J, et al. AMSTAR 2: a critical appraisal tool for systematic reviews that include randomised or non-randomised studies of healthcare interventions, or both. BMJ 2017; 358. 10.1136/bmj.j4008. 10.1136/bmj.j4008 PMC5833365 28935701

[26] Brouwers MC, Kho ME, Browman GP, Burgers JS, Cluzeau F, Feder G (2010). AGREE II: advancing guideline development, reporting and evaluation in health care. CMAJ Can Med Assoc J.

[27] Halsey EJ, Xing M, Stockley RC (2015). Acupuncture for joint symptoms related to aromatase inhibitor therapy in postmenopausal women with early-stage breast cancer: a narrative review. Acupunct Med.

[28] Dowling M, McDonagh B, Meade E. Arthralgia in breast cancer survivors: an integrative review of endocrine therapy. Oncol Nurs Forum 2017; 44. 10.1188/17.337-349.

[29] Bordeleau L, Pritchard K, Goodwin P, Loprinzi C (2007). Therapeutic options for the management of hot flashes in breast cancer survivors: an evidence-based review. Clin Ther.

[30] Ruan X, Mueck AO, Beer A-M, Naser B, Pickartz S (2019). Benefit-risk profile of black cohosh (isopropanolic Cimicifuga racemosa extract) with and without St John’s wort in breast cancer patients. Climacteric.

[31] Bae K, Yoo H-S, Lamoury G, Boyle F, Rosenthal DS, Oh B (2015). Acupuncture for aromatase inhibitor–induced arthralgia. Integr Cancer Ther.

[32] Chao L-F, Zhang AL, Liu H-E, Cheng M-H, Lam H-B, Lo SK (2009). The efficacy of acupoint stimulation for the management of therapy-related adverse events in patients with breast cancer: a systematic review. Breast Cancer Res Treat.

[33] Roberts K, Rickett K, Greer R, Woodward N (2017). Management of aromatase inhibitor induced musculoskeletal symptoms in postmenopausal early Breast cancer: A systematic review and meta-analysis. Crit Rev Oncol Hematol.

[34] Salehi A, Marzban M, Zadeh AR (2016). Acupuncture for treating hot flashes in breast cancer patients: an updated meta-analysis. Support Care Cancer.

[35] Tremblay A, Sheeran L, Aranda SK (2008). Psychoeducational interventions to alleviate hot flashes: a systematic review. Menopause.

[36] Finnegan-John J, Molassiotis A, Richardson A, Ream E (2013). A systematic review of complementary and alternative medicine interventions for the management of cancer-related fatigue. Integr Cancer Ther.

[37] Taylor S, Harley C, Ziegler L, Brown J, Velikova G (2011). Interventions for sexual problems following treatment for breast cancer: a systematic review. Breast Cancer Res Treat.

[38] Mazzarello S, Hutton B, Ibrahim MFK, Jacobs C, Shorr R, Smith S (2015). Management of urogenital atrophy in breast cancer patients: a systematic review of available evidence from randomized trials. Breast Cancer Res Treat.

[39] Chan CWH, Tai D, Kwong S, Chow KM, Chan DNS, Law BMH. The effects of pharmacological and non-pharmacological interventions on symptom management and quality of life among breast cancer survivors undergoing adjuvant endocrine therapy: a systematic review. Int J Environ Res Public Health 2020;17. 10.3390/ijerph17082950. 10.3390/ijerph17082950 PMC7215959 32344759

[40] Chen L, Lin C-C, Huang T-W, Kuan Y-C, Huang Y-H, Chen H-C (2017). Effect of acupuncture on aromatase inhibitor-induced arthralgia in patients with breast cancer: A meta-analysis of randomized controlled trials. Breast.

[41] Chien T-J, Liu C-Y, Chang Y-F, Fang C-J, Hsu C-H (2015). Acupuncture for treating aromatase inhibitor–related arthralgia in breast cancer: a systematic review and meta-analysis. J Altern Complement Med.

[42] Fritz H, Seely D, Flower G, Skidmore B, Fernandes R, Vadeboncoeur S (2013). Soy, Red Clover, and Isoflavones and Breast Cancer: A Systematic Review. PLoS ONE.

[43] Fritz H, Seely D, McGowan J, Skidmore B, Fernandes R, Kennedy DA (2014). Black Cohosh and Breast Cancer: A Systematic Review. Integr Cancer Ther.

[44] Pan Y, Yang K, Shi X, Liang H, Shen X, Wang R (2018). Clinical Benefits of Acupuncture for the Reduction of Hormone Therapy-Related Side Effects in Breast Cancer Patients: A Systematic Review. Integr Cancer Ther.

[45] Garcia MK, Graham-Getty L, Haddad R, Li Y, McQuade J, Lee RT (2015). Systematic review of acupuncture to control hot flashes in cancer patients: Acupuncture for Hot Flashes. Cancer.

[46] Lee MS, Kim K-H, Choi S-M, Ernst E (2009). Acupuncture for treating hot flashes in breast cancer patients: a systematic review. Breast Cancer Res Treat.

[47] Nahm N, Mee S, Marx G (2018). Efficacy of management strategies for aromatase inhibitor-induced arthralgia in breast cancer patients: a systematic review. Asia Pac J Clin Oncol.

[48] Yang GS, Kim HJ, Griffith KA, Zhu S, Dorsey SG, Renn CL (2017). Interventions for the Treatment of Aromatase Inhibitor-Associated Arthralgia in Breast Cancer Survivors: A Systematic Review and Meta-analysis. Cancer Nurs.

[49] Lu G, Zheng J, Zhang L (2020). The effect of exercise on aromatase inhibitor-induced musculoskeletal symptoms in breast cancer survivors: a systematic review and meta-analysis. Support Care Cancer.

[50] Boing L, Vieira M de CS, Moratelli J, Bergmann A, Guimarães AC de A. Effects of exercise on physical outcomes of breast cancer survivors receiving hormone therapy - A systematic review and meta-analysis. Maturitas 2020; 141: 71–81. 10.1016/j.maturitas.2020.06.022. 10.1016/j.maturitas.2020.06.022 33036706

[51] Li Y, Zhu X, Bensussan A, Li P, Moylan E, Delaney G (2016). Herbal Medicine for Hot Flushes Induced by Endocrine Therapy in Women with Breast Cancer: A Systematic Review and Meta-Analysis. Evid-Based Complement Altern Med.

[52] Liu L, Tan H, Yu S, Yin H, Baxter GD (2020). The effectiveness of tai chi in breast cancer patients: A systematic review and meta-analysis. Complement Ther Clin Pract.

[53] Johns C, Seav SM, Dominick SA, Gorman JR, Li H, Natarajan L (2016). Informing hot flash treatment decisions for breast cancer survivors: a systematic review of randomized trials comparing active interventions. Breast Cancer Res Treat.

[54] Kassab S, Cummings M, Berkovitz S, van Haselen R, Fisher P (2009). Homeopathic medicines for adverse effects of cancer treatments. Cochrane Database Syst Rev.

[55] Pan Y, Yang K, Shi X, Liang H, Zhang F, Lv Q (2015). Tai Chi Chuan Exercise for Patients with Breast Cancer: A Systematic Review and Meta-Analysis. Evid Based Complement Alternat Med.

[56] Rada G, Capurro D, Pantoja T, Corbalán J, Moreno G, Letelier LM (2010). Non-hormonal interventions for hot flushes in women with a history of breast cancer. Cochrane Database Syst Rev.

[57] Cramer H, Lauche R, Klose P, Lange S, Langhorst J, Dobos GJ (2017). Yoga for improving health-related quality of life, mental health and cancer-related symptoms in women diagnosed with breast cancer. Cochrane Database Syst Rev.

[58] Cramp F, Byron-Daniel J (2012). Exercise for the management of cancer-related fatigue in adults. Cochrane Database Syst Rev.

[59] Roberts KE, Rickett K, Feng S, Vagenas D, Woodward NE (2020). Exercise therapies for preventing or treating aromatase inhibitor-induced musculoskeletal symptoms in early breast cancer. Cochrane Database Syst Rev.

[60] Royal College of Obstetricians and Gynaecologists. Alternatives to HRT for the management of symptoms of the menopause. 2010.

[61] Runowicz CD, Leach CR, Henry NL, Henry KS, Mackey HT, Cowens-Alvarado RL (2016). American Cancer Society/American Society of Clinical Oncology Breast Cancer Survivorship Care Guideline. J Clin Oncol.

[62] National Cancer Institute. Hot flashes and night sweats (PDQ(R)) - Health professional version. Bethesda, MD: National Cancer Institute; 2019. Available from: https://www.cancer.gov/about-cancer/treatment/side-effects/hot-flashes-hp-pdq#_56

[63] Lyman GH, Greenlee H, Bohlke K, Bao T, DeMichele AM, Deng GE (2018). Integrative Therapies During and After Breast Cancer Treatment: ASCO Endorsement of the SIO Clinical Practice Guideline. J Clin Oncol.

[64] National Cancer Institute. Sleep disorders (PDQ(R)) - Health professional version. Bethesda, MD: National Cancer Institute; 2016. Available from: https://www.cancer.gov/about-cancer/treatment/side-effects/sleep-disorders-hp-pdq

[65] Nonhormonal management of menopause-associated vasomotor symptoms (2015). 2015 position statement of The North American Menopause Society. Menopause.

[66] National Cancer Institute. Fatigue (PDQ(R)) - Health professional version. Bethesda, MD: National Cancer Institute; 2017. Available from: https://www.cancer.gov/about-cancer/treatment/side-effects/fatigue/fatigue-hp-pdq

[67] Carter J, Lacchetti C, Andersen BL, Barton DL, Bolte S, Damast S (2018). Interventions to Address Sexual Problems in People With Cancer: American Society of Clinical Oncology Clinical Practice Guideline Adaptation of Cancer Care Ontario Guideline. J Clin Oncol.

[68] Bower JE, Bak K, Berger A, Breitbart W, Escalante CP, Ganz PA (2014). Screening, Assessment, and Management of Fatigue in Adult Survivors of Cancer: An American Society of Clinical Oncology Clinical Practice Guideline Adaptation. J Clin Oncol.

[69] National Comprehensive Cancer Network. Cancer-Related Fatigue. 2020. Available from: https://www.nccn.org/professionals/physician_gls/pdf/fatigue.pdf

[70] Faubion SS, Larkin LC, Stuenkel CA, Bachmann GA, Chism LA, Kagan R (2018). Management of genitourinary syndrome of menopause in women with or at high risk for breast cancer: consensus recommendations from The North American Menopause Society and The International Society for the Study of Women’s Sexual Health. Menopause N Y N.

[71] Cancer Australia. Management of menopausal symptoms in women with a history of breast cancer. Surry Hills, New South Wales, Australia: Cancer Australia; 2016. Available from: https://www.canceraustralia.gov.au/publications-and-resources/clinical-practice-guidelines/menopausal-guidelines

[72] Cancer Australia. Managing menopausal symptoms after breast cancer: a guide for women. Surry Hills, New South Wales, Australia: Cancer Australia; 2016. Available from: https://www.canceraustralia.gov.au/publications-and-resources/cancer-australia-publications/managing-menopausal-symptoms-after-breast-cancer-guide-women

[73] Cancer Australia. Guidance for the management of early breast cancer. Recommendations and practice points. Surry Hills, New South Wales, Australia: 2020. Available from: https://www.canceraustralia.gov.au/publications-and-resources/cancer-australia-publications/guidance-management-early-breast-cancer-recommendations-and-practice-points

[74] National Institute for Health and Care Excellence. Clinical knowledge summary: Tamoxifen - managing adverse effects. London, UK: 2014. Available from: https://cks.nice.org.uk/topics/tamoxifen-managing-adverse-effects/

[75] British Menopause Society, Association of Breast Surgeons. The diagnosis of the menopause and management of oestrogen deficiency symptoms and arthralgia in women treated for breast cancer. 2018. Available from: https://associationofbreastsurgery.org.uk/media/64908/11-bms-consensusstatement-the-diagnosis-of-the-menopause-and-management-of-oestrogen-deficiency-symptoms-and-arthralgia-in-women-treated-for-breast-cancer-for-abs-01c.pdf

[76] Alberta Health Services. Follow-up care for early stage breast cancer. 2015. Available from: https://www.albertahealthservices.ca/assets/info/hp/cancer/if-hp-cancer-guide-br013-early-stage-follow-up.pdf

[77] Fabi A, Bhargava R, Fatigoni S, Guglielmo M, Horneber M, Roila F (2020). Cancer-related fatigue: ESMO Clinical Practice Guidelines for diagnosis and treatment. Ann Oncol.

[78] Franzoi MA, Agostinetto E, Perachino M, Mastro LD, Azambuja E de, Vaz-Luis I, et al. Evidence-based approaches for the management of side-effects of adjuvant endocrine therapy in patients with breast cancer. Lancet Oncol 2021;0. 10.1016/S1470-2045(20)30666-5. 10.1016/S1470-2045(20)30666-5 33891888

[79] Brown JC, Winters-Stone K, Lee A, Schmitz KH (2012). Cancer, Physical Activity, and Exercise. Compr Physiol.

[80] de Rezende LFM, de Sá TH, Markozannes G, Rey-López JP, Lee I-M, Tsilidis KK (2018). Physical activity and cancer: an umbrella review of the literature including 22 major anatomical sites and 770 000 cancer cases. Br J Sports Med.

[81] Rock CL, Thomson C, Gansler T, Gapstur SM, McCullough ML, Patel AV (2020). American Cancer Society guideline for diet and physical activity for cancer prevention. CA Cancer J Clin.

[82] Irwin ML, Cartmel B, Gross CP, Ercolano E, Li F, Yao X (2015). Randomized exercise trial of aromatase inhibitor-induced arthralgia in breast cancer survivors. J Clin Oncol.

[83] Slade SC, Dionne CE, Underwood M, Buchbinder R (2016). Consensus on Exercise Reporting Template (CERT): Explanation and Elaboration Statement. Br J Sports Med.

[84] Burstein HJ, Lacchetti C, Anderson H, Buchholz TA, Davidson NE, Gelmon KA (2018). Adjuvant Endocrine Therapy for Women With Hormone Receptor-Positive Breast Cancer: ASCO Clinical Practice Guideline Focused Update. J Clin Oncol.

