# Supplementary material for: Strategies to self-manage side-effects of adjuvant endocrine therapy among breast cancer survivors: an umbrella review of empirical evidence and clinical guidelines
Source: J Cancer Surviv. 2021 Oct 18;16(6):1296–338. doi: 10.1007/s11764-021-01114-7 (PMC9630394; doi:10.1007/s11764-021-01114-7)
Supplement: Supplementary file 1 — Supplementary file1 (DOCX 36 kb) [file 11764_2021_1114_MOESM1_ESM.docx]

Figure 1. PRISMA Flowchart of systematic reviews included within umbrella review

Records identified through database searching
(n=906)

## Screening

## Included

## Eligibility

## Identification

Records after duplicates removed
(n=580)

Records screened
(n=580)

Records excluded
(n=510)

Full-text articles assessed for eligibility
(n=70)

Full-text articles excluded:

- Not a systematic review (n=20)
- Conference abstract (n=10)

- Does not discuss management or treatment of side effects (n=5)

- Involves a clinically prescribed intervention (n=7)

- An updated review has been performed and included (n=1)

- Does not discuss side effects from AET (n=2)

- Duplicate (n=1)

- Non-English language (n=1)

Reviews eligible (n=23)

Reviews included (n=33)

Backwards citation identified within systematic reviews (n=4)

Backwards citation identified within clinical guidelines (n=6)

Figure 2. PRISMA flowchart for guidelines included within umbrella review

Records identified through website searching
(n=1426)

## Screening

## Included

## Eligibility

## Identification

Records after duplicates removed
(n=953)

Records screened
(n=953)

Records excluded
(n=831)

Full guidelines assessed for eligibility
(n=122)

Full-text articles excluded:

- Not focussed on breast cancer patients (n=24)

- Not reviewing AET (n=3)

- Does not discuss management or treatment of side-effects (n=40)

- Does not discuss side-effects from AET (n=23)

- Only includes side-effects requiring medical management (n=2)

- Involves interventions requiring clinical oversight (n=3)

- Unavailable (n=8)

- Duplicate (n=2)

Guidelines included (n=18)

Backwards citation identified within clinical guidelines (n=1)
