# Supplementary material for: Strategies to self-manage side-effects of adjuvant endocrine therapy among breast cancer survivors: an umbrella review of empirical evidence and clinical guidelines
Source: J Cancer Surviv. 2021 Oct 18;16(6):1296–338. doi: 10.1007/s11764-021-01114-7 (PMC9630394; doi:10.1007/s11764-021-01114-7)
Supplement: Supplementary file 2 — Supplementary file2 (DOCX 56 kb) [file 11764_2021_1114_MOESM2_ESM.docx]

Update Search Strategies

In March 2019 we searched for systematic reviews examining the treatment of common side effects experienced by breast cancer patients being treated with tamoxifen or aromatase inhibitors. We updated and reran the searches in November 2020. The following databases were searched

| **Database** | **Date searched** |
| --- | --- |
| CINAHL (EBSCOHost) 1981 to present | 06-11-2020 |
| Cochrane Database of Systematic Reviews (Wiley): Issue 11 of 12, November 2020 | 06-11-2020 |
| Embase Classic+Embase (Ovid) 1947 to 2020 November 05 | 06-11-2020 |
| Ovid MEDLINE(R) and Epub Ahead of Print, In-Process & Other Non-Indexed Citations and Daily 1946 to November 05, 2020 | 06-11-2020 |
| Web of Science Core Collection: Citation Indexes (Clarivate Analytics) 1900 to present | 06-11-2020 |

## CINAHL (EBSCOHost) 1981 to present

Friday, November 06, 2020 4:58:50 PM

S61 S17 OR S59 60 [Limiters - Publication Type: Meta Analysis, Systematic Review]

S60 S17 OR S59 1,400

S59 S3 AND S11 AND S58 666

S58 S18 OR S19 OR S20 OR S21 OR S22 OR S23 OR S24 OR S25 OR S26 OR S27 OR S28 OR S29 OR S30 OR S31 OR S32 OR S33 OR S34 OR S35 OR S36 OR S37 OR S38 OR S39 OR S40 OR S41 OR S42 OR S43 OR S44 OR S45 OR S46 OR S47 OR S48 OR S49 OR S50 OR S51 OR S52 OR S53 OR S54 OR S55 OR S56 OR S57 469,091

S57 TI ( (vulva* or vagina* or vulvovaginal) N3 (atroph* or dry*) ) OR AB ( (vulva* or vagina* or vulvovaginal) N3 (atroph* or dry*) ) 889

S56 (MH "Vulvar Diseases") OR (MH "Vaginal Diseases") OR (MH "Dyspareunia") 3,067

S55 (MH "Body Weight Changes+") 124,641

S54 TI ( (taste or weight) N5 (change* or alter* or gain* or loss) ) OR AB ( (taste or weight) N5 (change* or alter* or gain* or loss) ) 48,236

S53 (MH "Taste Disorders+") 910

S52 TI ( "pins and needles" ) OR AB ( "pins and needles" ) 77

S51 TI ( paresthesia or dysthesia or tingl* ) OR AB ( paresthesia or dysthesia or tingl* ) 2,762

S50 (MH "Paresthesia") 1,184

S49 TI ( skin N4 (reaction* or sensation* or sensitiv*) ) OR AB ( skin N4 (reaction* or sensation* or sensitiv*) ) 2,610

S48 TI ( malaise or myalgia or arthralgia ) OR AB ( malaise or myalgia or arthralgia ) 4,397

S47 TI ( (joint or muscle or muscular) N3 (ache* or aching or pain* or sore* or tender* or symptom* or complain*) ) OR AB ( (joint or muscle or muscular) N3 (ache* or aching or pain* or sore* or tender* or symptom* or complain*) ) 13,724

S46 (MH "Muscle Pain") OR (MH "Joint Diseases") OR (MH "Arthralgia") 10,478

S45 TI ( hyperhidrosis or sweat* ) OR AB ( hyperhidrosis or sweat* ) 4,743

S44 (MH "Hyperhidrosis+") 740

S43 (MH "Climacteric") 769

S42 TI ( hot flash* or hot flush* ) OR AB ( hot flash* or hot flush* ) 2,401

S41 (MH "Hot Flashes") 2,348

S40 TI headache* OR AB headache* 27,372

S39 (MH "Headache") 13,896

S38 TI ( oedema or edema or swelling ) OR AB ( oedema or edema or swelling ) 32,407

S37 (MH "Edema") 8,111

S36 TI ( fluid* N2 (retention or retain* or accumulat*) ) OR AB ( fluid* N2 (retention or retain* or accumulat*) ) 1,271

S35 TI ( gas or bloat* or flatulence or nausea or nauseous* or vomit* ) OR AB ( gas or bloat* or flatulence or nausea or nauseous* or vomit* ) 43,951

S34 TI ( (abdominal or abdomen or gastrointestinal) N3 (pain* or discomfort or uncomfortable or complain*) ) OR AB ( (abdominal or abdomen or gastrointestinal) N3 (pain* or discomfort or uncomfortable or complain*) ) 14,625

S33 (MH "Signs and Symptoms, Digestive"+) 240

S32 (MH "Gastrointestinal Diseases") 7,889

S31 TI ( dizziness or dizzy or lightheaded* or light-headed* ) OR AB ( dizziness or dizzy or lightheaded* or light-headed* ) 6,113

S30 (MH "Dizziness") 2,762

S29 TI ( sleep* or drowsiness or drowsy or insomnia ) OR AB ( sleep* or drowsiness or drowsy or insomnia ) 69,850

S28 (MH "Sleep Disorders+") 39,702

S27 TI ( constipation or diarrhoea* or diarrhea* ) OR AB ( constipation or diarrhoea* or diarrhea* ) 24,496

S26 (MH "Constipation") OR (MH "Diarrhea") 16,268

S25 TI carpal tunnel OR AB carpal tunnel 3,070

S24 (MH "Carpal Tunnel Syndrome") 3,056

S23 TI ( asthenia or weakness or fatigue or tired* or lassitude or weary or weariness or exhaust* ) OR AB ( asthenia or weakness or fatigue or tired* or lassitude or weary or weariness or exhaust* ) 71,796

S22 (MH "Fatigue") OR (MH "Cancer Fatigue") 19,814

S21 (MH "Asthenia") 212

S20 TI ( appetite or anorexia or anoretic or anorectic ) OR AB ( appetite or anorexia or anoretic or anorectic ) 14,650

S19 (MH "Anorexia") 1,768

S18 (MH "Appetite") 3,473

S17 S3 AND S11 AND S16 1,002

S16 S12 OR S13 OR S14 OR S15 187,257

S15 TI toxicit* OR AB toxicit* 44,053

S14 TI ( adverse N3 (event* or effect* or reaction*) ) OR AB ( adverse N3 (event* or effect* or reaction*) ) 108,642

S13 TI side-effect* OR AB side-effect* 44,329

S12 (MH "Adverse Drug Event") 13,913

S11 S4 OR S5 OR S6 OR S7 OR S8 OR S9 OR S10 9,093

S10 TI ( anastrozole or letrozole or exemestane ) OR AB ( anastrozole or letrozole or exemestane ) 1,547

S9 TI aromatase inhibitor* OR AB aromatase inhibitor* 1,880

S8 (MH "Aromatase Inhibitors+") 2,178

S7 TI selective oestrogen receptor modulator* OR AB selective oestrogen receptor modulator* 612

S6 TI selective estrogen receptor modulator* OR AB selective estrogen receptor modulator* 667

S5 TI ( tamoxifen or SERM or SERMS ) OR AB ( tamoxifen or SERM or SERMS ) 3,965

S4 (MH "Selective Estrogen Receptor Modulators+") 5,440

S3 S1 OR S2 104,305

S2 TI ( (breast* or mammar*) N4 (cancer* or carcinoma* or neoplas* or adenocarcinoma* or malignan* or tumo?r* or sarcoma*) ) OR AB ( (breast* or mammar*) N4 (cancer* or carcinoma* or neoplas* or adenocarcinoma* or malignan* or tumo?r* or sarcoma*) ) 81,076

S1 (MH "Breast Neoplasms+") 84,219

## Cochrane Library

Search Name: Cancer Side Effects

Date Run: 06/11/2020 21:49:38

Comment: draft 01-03-19 - correction made 06-11-20

- Cochrane Database of Systematic Reviews (Wiley): Issue 11 of 12, November 2020 (n=19)
- Cochrane Central Register of Controlled Trials (Wiley): Issue 11 of 12, November 2020 (n=2544)

ID Search Hits

#1 MeSH descriptor: [Breast Neoplasms] explode all trees 13068

#2 ((breast* or mammar*) NEAR/4 (cancer* or carcinoma* or neoplas* or adenocarcinoma* or malignan* or tumor* or tumour* or sarcoma*)):ti,ab,kw 37475

#3 #1 or #2 37476

#4 MeSH descriptor: [Tamoxifen] explode all trees 2296

#5 tamoxifen:ti,ab,kw 4901

#6 (SERM or SERMS):ti,ab,kw 296

#7 selective estrogen receptor modulator*:ti,ab,kw 818

#8 selective oestrogen receptor modulator*:ti,ab,kw 818

#9 MeSH descriptor: [Aromatase Inhibitors] explode all trees 686

#10 "aromatase inhibitor*":ti,ab,kw 1483

#11 (anastrozole or letrozole or exemestane):ti,ab,kw 3459

#12 #5 or #6 or #7 or #8 or #9 or #10 or #11 8395

#13 MeSH descriptor: [Drug-Related Side Effects and Adverse Reactions] explode all trees 3595

#14 "side-effect*":ti,ab,kw 90050

#15 (adverse NEAR/3 (event* or effect* or reaction*)):ti,ab,kw 280173

#16 toxicit*:ti,ab,kw 45490

#17 #13 or #14 or #15 or #16 329661

#18 #3 and #12 and #17 2283

#19 MeSH descriptor: [Appetite] explode all trees 1446

#20 MeSH descriptor: [Anorexia] explode all trees 386

#21 (appetite or anorexia or anoretic or anorectic):ti,ab,kw 14533

#22 MeSH descriptor: [Asthenia] explode all trees 78

#23 MeSH descriptor: [Fatigue] explode all trees 3698

#24 (asthenia or weakness or fatigue* or tired* or lassitude or weary or weariness or exhaust*):ti,ab,kw 45440

#25 MeSH descriptor: [Carpal Tunnel Syndrome] explode all trees 694

#26 "carpal tunnel":ti,ab,kw 1552

#27 MeSH descriptor: [Constipation] explode all trees 1714

#28 MeSH descriptor: [Diarrhea] explode all trees 3507

#29 (constipation or diarrhoea* or diarrhea*):ti,ab,kw 36688

#30 (sleep* or drowsiness or drowsy or insomnia):ti,ab,kw 47338

#31 MeSH descriptor: [Sleep Initiation and Maintenance Disorders] explode all trees 2449

#32 MeSH descriptor: [Dizziness] explode all trees 742

#33 (dizziness or dizzy or lightheaded* or light-headed*):ti,ab,kw 13663

#34 MeSH descriptor: [Gastrointestinal Diseases] this term only 1635

#35 MeSH descriptor: [Signs and Symptoms, Digestive] explode all trees 15073

#36 ((abdominal or abdomen or gastrointestinal) NEAR/3 (pain* or discomfort or uncomfortable or complain*)):ti,ab,kw 15047

#37 (gas or bloat* or flatulence or nausea or nauseous* or vomit*):ti,ab,kw 72513

#38 (fluid* near/2 (retention or retain* or accumulat*)):ti,ab,kw 1087

#39 MeSH descriptor: [Edema] explode all trees 1782

#40 (oedema or edema or swelling):ti,ab,kw 25097

#41 MeSH descriptor: [Headache] explode all trees 2387

#42 headache*:ti,ab,kw 31688

#43 MeSH descriptor: [Hot Flashes] explode all trees 872

#44 (hot flash* or hot flush*):ti,ab,kw 3549

#45 MeSH descriptor: [Climacteric] this term only 329

#46 MeSH descriptor: [Hyperhidrosis] explode all trees 258

#47 (hyperhidrosis or sweat*):ti,ab,kw 4279

#48 MeSH descriptor: [Joint Diseases] this term only 651

#49 MeSH descriptor: [Myalgia] explode all trees 462

#50 MeSH descriptor: [Arthralgia] explode all trees 1792

#51 ((joint or muscle or muscular) NEAR/3 (ache* or aching or pain* or sore* or tender* or symptom* or complain*)):ti,ab,kw 10193

#52 (malaise or myalgia or arthralgia):ti,ab,kw 9670

#53 (skin Near/4 (reaction* or sensation* or sensitiv*)):ti,ab,kw 3790

#54 MeSH descriptor: [Paresthesia] explode all trees 273

#55 (paresthesia or dysthesia or tingl*):ti,ab,kw 4021

#56 "pins and needles":ti,ab,kw 47

#57 MeSH descriptor: [Taste Disorders] explode all trees 140

#58 ((taste or weight) NEAR/5 (change* or alter* or gain* or loss)):ti,ab,kw 36731

#59 MeSH descriptor: [Body Weight Changes] explode all trees 8714

#60 ((vulva* or vagina* or vulvovaginal) NEAR/3 (atroph* or dry*)):ti,ab,kw 984

#61 MeSH descriptor: [Dyspareunia] this term only 201

#62 MeSH descriptor: [Atrophic Vaginitis] explode all trees 28

#63 MeSH descriptor: [Vulvar Diseases] explode all trees 648

#64 MeSH descriptor: [Vaginal Diseases] explode all trees 1302

#65 {OR #19-#64} 257219

#66 #3 and #12 and #65 1122

#67 #66 or #18 2563

## Embase Classic+Embase (Ovid) <1947 to 2020 November 05>

Search Date: 6 November 2020

--------------------------------------------------------------------------------

1 ((breast* or mammar*) adj4 (cancer* or carcinoma* or neoplas* or adenocarcinoma* or malignan* or tumo?r* or sarcoma*)).tw,kw. (534424)

2 exp *breast cancer/ (320939)

3 1 or 2 [breast cancer] (559550)

4 *tamoxifen/ (18550)

5 tamoxifen.tw. (34335)

6 SERM?.tw. (3364)

7 *selective estrogen receptor modulator/ (2006)

8 selective estrogen receptor modulator*.tw. (4154)

9 selective oestrogen receptor modulator*.tw. (408)

10 exp *aromatase inhibitor/ (11074)

11 "aromatase inhibitor*".tw. (12057)

12 (anastrozole or letrozole or exemestane).tw. (8840)

13 or/4-12 [SERMs & AI treatment] (56615)

14 *adverse drug reaction/ (123437)

15 side-effect*.tw,kw. (397572)

16 (adverse adj3 (event* or effect* or reaction*)).tw,kw. (651606)

17 toxicit*.tw,kw. (614867)

18 or/14-17 [generic AEs] (1600819)

19 3 and 13 and 18 (6220)

20 limit 19 to ((meta analysis or "systematic review") and "reviews (best balance of sensitivity and specificity)") (298)

21 (appetite or anorexia or anoretic or anorectic).tw,kw. (90190)

22 appetite disorder/ or decreased appetite/ or increased appetite/ or "loss of appetite"/ (27802)

23 *asthenia/ (1704)

24 *fatigue/ or exp *exhaustion/ or exp lassitude/ (26153)

25 (asthenia or weakness or fatigue* or tired* or lassitude or weary or weariness or exhaust*).tw,kw. (355792)

26 *carpal tunnel syndrome/ (9523)

27 carpal tunnel.tw,kw. (13828)

28 *constipation/ or *diarrhea/ (49637)

29 (constipation or diarrhoea* or diarrhea*).tw,kw. (207847)

30 *sleep/ or *drowsiness/ (55892)

31 (sleep* or drowsiness or drowsy or insomnia).tw,kw. (323107)

32 *dizziness/ (2343)

33 (dizziness or dizzy or lightheaded* or light-headed*).tw,kw. (36948)

34 *abdominal disease/ or *abdominal discomfort/ or *bloating/ or *flatulence/ (3120)

35 *gastrointestinal disease/ (56346)

36 ((abdominal or abdomen or gastrointestinal) adj3 (pain* or discomfort or uncomfortable or complain*)).tw,kw. (124224)

37 (gas or bloat* or flatulence or nausea or nauseous* or vomit*).tw,kw. (531537)

38 exp *"nausea and vomiting"/ (37807)

39 (fluid* adj2 (retention or retain* or accumulat*)).tw,kw. (11022)

40 *peripheral edema/ (372)

41 (oedema or edema or swelling).tw,kw. (350276)

42 *headache/ (33452)

43 headache*.tw,kw. (146989)

44 *hot flush/ (2478)

45 (hot flash* or hot flush*).tw,kw. (7591)

46 *climacterium/ (4397)

47 *hyperhidrosis/ (3230)

48 (hyperhidrosis or sweat*).tw,kw. (43697)

49 *arthropathy/ (13230)

50 *myalgia/ or *arthralgia/ (10065)

51 ((joint or muscle or muscular) adj3 (ache* or aching or pain* or sore* or tender* or symptom* or complain*)).tw,kw. (50472)

52 *malaise/ (760)

53 (malaise or myalgia or arthralgia).tw,kw. (34581)

54 (skin adj4 (reaction* or sensation* or sensitiv*)).tw,kw. (31266)

55 *skin tingling/ or *paresthesia/ (3348)

56 (paresthesia or dysthesia or tingl*).tw,kw. (13975)

57 "pins and needles".tw,kw. (337)

58 exp *taste disorder/ (1821)

59 ((taste or weight) adj5 (change* or alter* or gain* or loss)).tw,kw. (291587)

60 *weight gain/ or *weight loss/ or *weight change/ (16955)

61 ((vulva* or vagina* or vulvovaginal) adj3 (atroph* or dry*)).tw,kw. (3867)

62 *dyspareunia/ or *vagina atrophy/ (2209)

63 or/21-62 [specific symptoms] (2348039)

64 3 and 13 and 63 [specific side effects breast ca tmt] (2960)

65 limit 64 to (meta analysis or "systematic review") (119)

66 20 or 65 (340)

67 ("Search filter*" or "search strateg*" or "literature search*").tw. (86922)

68 (Literature review* or scoping review* or synthesis or meta-analys* or "meta analysis").ti. (632805)

69 ((systematic or narrative or critical) adj2 review*).ti. (187461)

70 67 or 68 or 69 (800509)

71 64 and 70 (74)

72 66 or 71 (364)

## Ovid MEDLINE(R) and Epub Ahead of Print, In-Process & Other Non-Indexed Citations and Daily <1946 to November 05, 2020>

Includes Ovid MEDLINE(R) 1946 to October Week 5 2020

Search Date: 6 November 2020

--------------------------------------------------------------------------------

1 exp Breast Neoplasms/ (295673)

2 ((breast* or mammar*) adj4 (cancer* or carcinoma* or neoplas* or adenocarcinoma* or malignan* or tumo?r* or sarcoma*)).tw,kw. (362149)

3 1 or 2 [breast cancer] (421144)

4 exp Tamoxifen/ (21327)

5 tamoxifen.tw. (23020)

6 SERM?.tw. (2196)

7 selective estrogen receptor modulator*.tw. (3037)

8 selective oestrogen receptor modulator*.tw. (312)

9 exp aromatase inhibitors/ (8926)

10 "aromatase inhibitor*".tw. (7449)

11 anastrozole/ or letrozole/ (3111)

12 (anastrozole or letrozole or exemestane).tw. (4742)

13 or/4-12 [SERMs & AI treatment] (40521)

14 "Drug-Related Side Effects and Adverse Reactions"/ (32641)

15 side-effect*.tw,kf. (258419)

16 (adverse adj3 (event* or effect* or reaction*)).tw,kf. (406636)

17 toxicit*.tw,kw. (410619)

18 or/14-17 [generic Adverse effects] (1003866)

19 3 and 13 and 18 (3841)

20 appetite/ (7825)

21 anorexia/ (5003)

22 (appetite or anorexia or anoretic or anorectic).tw,kf. (57482)

23 Asthenia/ (1682)

24 fatigue/ (29095)

25 (asthenia or weakness or fatigue* or tired* or lassitude or weary or weariness or exhaust*).tw,kf. (215499)

26 Carpal Tunnel Syndrome/ (8677)

27 carpal tunnel.tw,kf. (10481)

28 constipation/ or diarrhea/ (60846)

29 (constipation or diarrhoea* or diarrhea*).tw,kf. (130784)

30 sleepiness/ (380)

31 (sleep* or drowsiness or drowsy or insomnia).tw,kf. (202718)

32 "Sleep Initiation and Maintenance Disorders"/ (13439)

33 Dizziness/ (5432)

34 (dizziness or dizzy or lightheaded* or light-headed*).tw,kf. (20438)

35 Gastrointestinal Diseases/ (39281)

36 exp "Signs and Symptoms, Digestive"/ (154454)

37 ((abdominal or abdomen or gastrointestinal) adj3 (pain* or discomfort or uncomfortable or complain*)).tw,kf. (69766)

38 (gas or bloat* or flatulence or nausea or nauseous* or vomit*).tw,kf. (379564)

39 (fluid* adj2 (retention or retain* or accumulat*)).tw,kf. (7477)

40 edema/ (40238)

41 (oedema or edema or swelling).tw,kf. (223469)

42 headache/ (27974)

43 headache*.tw,kf. (86654)

44 Hot Flashes/ (3255)

45 (hot flash* or hot flush*).tw,kf. (4782)

46 Climacteric/ (4880)

47 exp Hyperhidrosis/ (3738)

48 (hyperhidrosis or sweat*).tw,kf. (26444)

49 Joint Diseases/ (24694)

50 Myalgia/ or Arthralgia/ (10217)

51 ((joint or muscle or muscular) adj3 (ache* or aching or pain* or sore* or tender* or symptom* or complain*)).tw,kf. (30092)

52 (malaise or myalgia or arthralgia).tw,kf. (19351)

53 (skin adj4 (reaction* or sensation* or sensitiv*)).tw,kf. (18360)

54 Paresthesia/ (5945)

55 (paresthesia or dysthesia or tingl*).tw,kf. (8078)

56 "pins and needles".tw,kf. (152)

57 exp taste disorders/ (2330)

58 ((taste or weight) adj5 (change* or alter* or gain* or loss)).tw,kf. (187841)

59 weight gain/ or weight loss/ (66693)

60 ((vulva* or vagina* or vulvovaginal) adj3 (atroph* or dry*)).tw,kf. (2095)

61 Dyspareunia/ or Vulvar Diseases/ or Vaginal Diseases/ or Atrophic Vaginitis/ (9370)

62 or/20-61 [specific symptoms] (1669575)

63 3 and 13 and 62 [specific side effects breast ca tmt] (1676)

64 19 or 63 (4636)

65 limit 64 to (meta analysis or "systematic review") (134)

66 ("Search filter*" or "search strateg*" or "literature search*").tw. (69533)

67 (Literature review* or scoping review* or synthesis or meta-analys* or "meta analysis").ti. (469859)

68 ((systematic or narrative or critical) adj2 review*).ti. (155404)

69 66 or 67 or 68 (608462)

70 64 and 69 (169)

71 65 or 70 (205)

## Web of Science Core Collection: Citation Indexes (Clarivate Analytics) 1900 to present

Search date: 6 November 2020

- Science Citation Index Expanded (SCI-EXPANDED) --1900-present
- Social Sciences Citation Index (SSCI) --1900-present
- Arts & Humanities Citation Index (A&HCI) --1975-present
- Conference Proceedings Citation Index- Science (CPCI-S) --1990-present
- Conference Proceedings Citation Index- Social Science & Humanities (CPCI-SSH) --1990-present
- Emerging Sources Citation Index (ESCI) --2015-present

Data last updated: 2020-11-05

# 40 258 #39 AND #38

# 39 5,239 #35 OR #12

# 38 1,604,444 #37 OR #36

# 37 71,911 TOPIC: ("Search filter*" or "search strateg*" or "literature search*")

# 36 1,560,584 TITLE: (Literature review* or systematic near/2 review* or narrative near/2 review* or critical near/2 review* or scoping review* or synthesis or meta-analys* or "meta analysis")

# 35 2,063 #34 AND #7 AND #1

# 34 3,166,373 #33 OR #32 OR #31 OR #30 OR #29 OR #28 OR #27 OR #26 OR #25 OR #24 OR #23 OR #22 OR #21 OR #20 OR #19 OR #18 OR #17 OR #16 OR #15 OR #14 OR #13

# 33 3,988 TOPIC: (Dyspareunia)

# 32 2,177 TS=((vulva* or vagina* or vulvovaginal) NEAR/3 (atroph* or dry*) )

# 31 282,899 TS=((taste or weight) NEAR/5 (change* or alter* or gain* or loss or disorder*) )

# 30 140 TOPIC: ("pins and needles")

# 29 9,601 TS=(paresthesia or dysthesia or tingl*)

# 28 17,498 TOPIC: (skin NEAR/4 (reaction* or sensation* or sensitiv*) )

# 27 19,389 TOPIC: (malaise or myalgia or arthralgia)

# 26 34,664 TOPIC: ((joint or muscle or muscular) NEAR/3 (ache* or aching or pain* or sore* or tender* or symptom* or complain*) )

# 25 33,218 TOPIC: (hyperhidrosis or sweat* or climacteric)

# 24 5,531 TOPIC: ("hot flash*" or "hot flush*")

# 23 82,901 TOPIC: (headache*)

# 22 283,140 TS=(oedema or edema or swelling)

# 21 8,014 TOPIC: (fluid* NEAR/2 (retention or retain* or accumulat*) )

# 20 1,551,753 TOPIC: (gas or bloat* or flatulence or nausea or nauseous* or vomit*)

# 19 55,535 TOPIC: ((abdominal or abdomen or gastrointestinal) NEAR/3 (pain* or discomfort or uncomfortable or complain*) )

# 18 17,482 TOPIC: (dizziness or dizzy or lightheaded* or light-headed*)

# 17 276,160 TS=(sleep* or drowsiness or drowsy or insomnia)

# 16 129,353 TOPIC: (constipation or diarrhoea* or diarrhea*)

# 15 11,739 TOPIC: ("carpal tunnel")

# 14 522,120 TOPIC: (asthenia or weakness or fatigue* or tired* or lassitude or weary or weariness or exhaust*)

# 13 68,460 TOPIC: (appetite or anorexia or anoretic or anorectic)

# 12 4,118 #11 AND #7 AND #1

# 11 1,110,467 #10 OR #9 OR #8

# 10 575,367 TOPIC: (toxicit*)

# 9 414,142 TOPIC: (adverse NEAR/3 (event* or effect* or reaction*) )

# 8 210,629 TOPIC: ("side-effect*")

# 7 4 6,349 #6 OR #5 OR #4 OR #3 OR #2

# 6 8,474 TOPIC: (anastrozole or letrozole or exemestane)

# 5 10,463 TOPIC: ("aromatase inhibitor*")

# 4 327 TOPIC: ("selective oestrogen receptor modulator*")

# 3 3,232 TOPIC: ("selective estrogen receptor modulator*")

# 2 35,536 TOPIC: (tamoxifen or SERM or SERMS)

# 1 589,927 TOPIC: ((breast* or mammar*) NEAR/4 (cancer* or carcinoma* or neoplas* or adenocarcinoma* or malignan* or tumor* or tumour* or sarcoma*) )

# Guidelines search results & strategies Date: 13 November 2020

In July 2019 we performed a complementary website search for guidelines on the use of aromatase inhibitors or tamoxifen, or treating specific side-effects. The searches were rerun in November 2019 in the following guideline and cancer websites.

| **Website** | **Date searched** |
| --- | --- |
| American Society for Clinical Oncology (ASCO) <https://www.asco.org/research-guidelines/quality-guidelines/guidelines> | 12-11-20 |
| European Society of Medical Oncology (ESMO)  <https://www.esmo.org/> | 13-11-20 |
| CPG Infobase: Clinical Practice Guideline  <https://joulecma.ca/cpg/homepage#_ga=2.207853850.989988427.1605201906-936946155.1605201906> | 12-11-20 |
| International Guidelines Library (GIN) <https://www.g-i-n.net/library/international-guidelines-library/international-guidelines-library> | 12-11-20 |
| National Comprehensive Cancer Network (NCCN)  <https://www.nccn.org/professionals/physician_gls/default.aspx> | 12-11-20 |
| NIH National Cancer Institute <https://www.cancer.gov/about-cancer/treatment> | 13-11-20 |
| NHMRC Australian Clinical Practice Guidelines  <https://www.clinicalguidelines.gov.au/> | 12-11-20 |
| NICE Evidence  <https://www.evidence.nhs.uk/> | 11-11-20 |
| NIH National Cancer Institute (NCI) |  |
| SIGN  <https://www.sign.ac.uk/our-guidelines> | 12-11-20 |
| TRIP database  <https://www.tripdatabase.com/> | 13-11-20 |

All searches have used the basic all website search feature unless otherwise specified

## Adjuvant hormonal therapies

| **Date** | **website** | **Refs (#downloaded/#retrieved)** | **Time taken (mins)** |
| --- | --- | --- | --- |
| 9/11/20 | **NICE Evidence:** <https://www.evidence.nhs.uk/>  **Guidelines filter**  Cancer AND tamoxifen  Cancer AND SERM*  Cancer AND "selective estrogen receptor modulator*"  cancer AND "selective oestrogen receptor modulator*"  cancer AND "aromatase inhibitor*"  cancer AND (anastrozole OR letrozole OR exemestane) | **217 deduplicated to 130**  83  28  3  8  52  43 | 20 |
| 9/11/20 | **SIGN** <https://www.sign.ac.uk/our-guidelines.html>  aromatase inhibitor*  selective estrogen receptor modulator*  selective oestrogen receptor modulator*  (tamoxifen OR SERM OR anastrozole OR letrozole OR exemestane) *all searched individually all had 0 hits* | 1/6 records retrieved was relevant  ½ relevant  0/2  0/2 (same as above)  All 0 | 20  Only downloaded relevant guidelines. These were not added again if already found by previous searches as manually downloaded |
| 9/11/20 | **International GL Lib GIN**  <https://guidelines.ebmportal.com/>  tamoxifen  aromatase inhibitor*  selective estrogen receptor modulator*  (selective oestrogen receptor modulator* OR SERM OR anastrozole OR letrozole OR exemestane) these were *all searched individually each gave 0* | 1  1  0 | 10  2 refs. These were found in 2019 original search |
| 11/11/20 | **TRIP database**  [**https://www.tripdatabase.com/**](https://www.tripdatabase.com/)  use guidelines filter  “breast cancer” AND tamoxifen  “breast cancer” AND SERM  “breast cancer” AND (“Selective estrogen receptor modulator*”OR “Selective oestrogen receptor modulator*”)  “breast cancer AND “Aromatase inhibitor*”  Cancer AND (Anastrozole OR Letrozole OR exemestane) | **81 refs total added to d/b**  (14 new in 2020 update)  178  138  69  121  110 | 2.5 hrs  As downloading individually restricted to “breast cancer” and unique and relevant guidelines not found in other searches. Only the most current GL was added |
| 9/11/20 | **CPG Infobase: Clinical Practice Guideline**  [**https://joulecma.ca/cpg/homepages**](https://joulecma.ca/cpg/homepages)  tamoxifen  (aromatase inhibitor* OR selective estrogen receptor modulator* OR  selective oestrogen receptor modulator OR SERM OR anastrozole OR letrozole OR exemestane) *all searched individually gave 0 hits* | 1 (already in database) | 15  Did not include full text in search |
| 9/11/20 | NHMRC Australian CPGs  <https://www.clinicalguidelines.gov.au/>  (tamoxifen OR aromatase inhibitor* OR selective estrogen receptor modulator* OR selective oestrogen receptor modulator OR SERM OR anastrozole OR letrozole OR exemestane) *all searched individually* | 0 | 5 |
| 11/11/20 | National Comprehensive Cancer Network: Clinical Practice Guidelines in Oncology  <https://www.nccn.org/professionals/physician_gls/default.aspx>  tamoxifen  (aromatase inhibitor* OR selective estrogen receptor modulator* OR selective oestrogen receptor modulator OR SERM OR anastrozole OR letrozole OR exemestane) *all searched individually* | 16  (3 in 2020 update) | 30  Only selected Guidelines/guidance for patients./ professionals relating to cancer and uniuq to search term breast cancer |
| 9/11/20 | American Society for Clinical Oncology: ASCO Guidelines <https://www.asco.org/research-guidelines/quality-guidelines/guidelines>  Tamoxifen  aromatase inhibitor/s  SERM  anastrozole  exemestane  (selective estrogen receptor modulator/s OR selective estrogen receptor modulator/s OR letrozole) *all searched individually* | 2/2  3/3 (2 in update)  3/3 (1 “”)  0/1 (already added above)  0/2 (1 in update already added)  0 (unique or relevant) | 25 mins  Searched for terms and filtered by *guideline* |
| 9/11/20 | ESMO <https://www.esmo.org/>  *Filtered by “Guideline”*  *Aromatase inhibitor**  selective estrogen receptor modulator* (same results for Oestrogen)  (tamoxifen OR SERM OR anastrozole or letrozole or exemestane) *all searched individually* | **17/**  16/17  1/10 (unique or relevant)  0 (unique or relevant) | 25 min  Site searches US/UK spelling of oestrogen/estrogen  Majority 9/10 of these refs found by aromatase search |
| 9/11/20 | NIH National Cancer Institute  <https://www.cancer.gov/about-cancer/treatment>  Searches publications PDQ series | 4 (O added to update) | 15 |

## Specific cancer side effects

| **Date** | **website** | **Refs (#downloaded/#retrieved)** | **Time taken**  **(mins)** |
| --- | --- | --- | --- |
| 11/11/20 | **NICE Evidence:** <https://www.evidence.nhs.uk/>  **Guidelines filter**  Sorted by relevance  Cancer AND (arthralgia OR “joint pain” OR “joint disorder*” OR “joint symptom*”)  Cancer AND (fatigue or insomnia)  cancer AND (hot flash* OR hot flush*)  Cancer AND nausea  Cancer AND “pain management”  cancer AND ( bloat* or "gastrointestinal discomfort" or "gastrointestinal pain" or "gastrointestinal symptoms” or “gastrointestinal problems”)  cancer AND ( "abdominal discomfort" or "abdominal pain" or "abdominal symptoms")  cancer AND (vulva or vagina* or vulvovaginal) AND (atrophy or dry* or pain) | **Note**: this search was very crude relates more to condition as a side effect rather than treating it. No adj etc possible to refine further  **758 dedup in grp to 496**  8/145  200/630  50/132  50/80 (could have been 20)  200/635  50/207  50/224 | 2:00  Long time refining search for a sensible number  ** downloaded first 50 or 100 records (sorted by relevance as majority after this were not cancer) |
| 12/11/20 | **SIGN** <https://www.sign.ac.uk/our-guidelines>  Individual searches not v relevant. Scanned list of current, proposed and in development guideline) | 2 ( 0 IN UPDATE) | 30  Only downloaded relevant – most not applicable |
| 12/11/20 | **International GL Lib GIN**  <https://www.g-i-n.net/library/international-guidelines-library/international-guidelines-library>  cancer AND joint  cancer AND fatigue  cancer AND nausea  cancer AND pain  cancer AND gastrointestinal  cancer AND vaginal  (Cancer AND arthralgia/ insomnia/ hot flush*/hot flash*/  Cancer and vagina*/vulva/vulvovaginal  Cancer AND bloat*/abdominal  *all searched individually* | 21 - 2 Added to EN  0/1  1/3 (non English/already have)  0/1 (already have)  2/5 (non English/already have)  0/9 (not relevant)  1/2 (not laoded as already had) | 0..75  Only downloaded unique articles in English |
| 13/11/20 | **TRIP database**  [**https://www.tripdatabase.com/**](https://www.tripdatabase.com/)  **use guidelines filter Sorted by relevance)**  **Decided to redo using PICO feature in TRIP this searches (title:x)(title: y)**  **Checked GL only**  Pop: cancer AND Outcome: (arthralgia or joint pain or joint disorder* or joint symptom*)  Pop: cancer AND Outcome: (insomnia or fatigue)  Pop: cancer AND Outcome: (hot flash* or hot flush*)  Pop: cancer AND Outcome: Pain  Pop: cancer AND Outcome: nausea  Cancer AND insomnia  Pop: cancer AND Outcome: (bloat* or gastrointestinal or abdominal)  Pop: cancer AND Outcome: (vulva or vagina* or vulvovaginal) | **Total = 15 ( 5 in update)**  0  0/1 (got already!)  0/1 (got already!)  0/0  17/42 (17 suitable 10 added)  4/4 (only 1 unique in 2020)  0  0/5 (  0/3 (all not relv) | **1 hr plus +1.5 hr* (*computer hanging issues)**  Manual entry only added those not already in database  Only selected pain manageable at home  30  NB Only selected relevant and unique records |
| 12/11/20 | **CPG Infobase: Clinical Practice Guideline**  <https://joulecma.ca/cpg/homepage#_ga=2.207853850.989988427.1605201906-936946155.1605201906>  cancer fatigue  cancer nausea  cancer pain  cancer vagina*  cancer vulva  cancer gastrointestinal  Cancer arthralgia/joint pain”/“joint disorder*/ “joint symptom*” /insomnia/hot flush*/hot flash*/ vulva/vulvovaginal/ abdominal/gastrointestinal)  *all searched individually* | 18*/31 (0 unique in update)  3  ¾  2/9 (suitable or GL)  0/9 (rlevant)  0/1(not relevant)  0/5(not relvant) | 30  Did not include full text in search. Only selected English GL  *2019 search included FT which explains 18 hits when only 8 relevant were detected in the 2020 update |
| 12/11/20 | NHMRC Australian CPGs  <https://www.clinicalguidelines.gov.au/>  cancer  pain  *all searched individually*  arthralgia/joint pain/joint symptom*/joint disorder/fatigue/nausea/hot flash*/hot flush*/bloat/abdominal/gastrointestinal/vulva/vagina/vulvovaginal | 0  0/8  0/1 (not relevant)  0 | 15 |
| 12/11/20 | National Comprehensive Cancer Network: Clinical Practice Guidelines in Oncology  <https://www.nccn.org/professionals/physician_gls/default.aspx>  *Searched the list of guidelines listed in the supportive care section of resources* | 6 (4 unique in 2020) | 15 mins  Professional guidelines were a locked resource – link to section only  Also linked to patient resources section which can be accessed |
| 12/11/20 | American Society for Clinical Oncology: ASCO Guidelines  <https://www.asco.org/research-guidelines/quality-guidelines/guidelines>  *Searched the list of guidelines listed in the* supportive care *and* patient & survivor car*e sections of resources plus key word searches filtered by guideline:*  Fatigue  pain  nausea  gastrointestinal  abdominal  vulva*  vagina*  Arthralgia/ Joint pain/disorder*/symptom*/ Hot flush*/ flash*/Bloat*/vagina*/vulvovaginal | 7 (4 in update)  2/2  3/5  1/1  0/3  0/2  1/1  ½  All 0 | *40 mins* |
| 13/11/20 | ESMO <https://www.esmo.org/>  *Checked supportive & palliative care a*nd patient guide sections only | 6 (3 unique in 2020) | 10 |
| 13/11/20 | NIH National Cancer Institute  <https://www.cancer.gov/about-cancer/treatment/side-effects>  Searched publications PDQ series *supportive & Palliative care section only*  Selected GL with refs | 7 (1 unique in 2020 | 15 |

## Total hits = 1426 (771 added in 2020 update)

## De-duplicated Endnote library contains: 916 (261 added in 2020)

(301 specific drugs & 615 side effects; 2020 update contained 90 drugs and 171side effects

NOTES:

- Total searching time: 13.6 hrs (ca 2 days 2019 ) and 9.5hrs (2020) majority was dealing with TRIP for manual downloading (see options for Macro with JW) and NHS Evidence – both which pulled up a lot of irrelevant material with default F/T searching
- Only unique hits in 2020 were added from the websites requiring manual individual download (all bar NHS evidence) so it was difficult to accurately estimate total number of hits in some databases

*** 41 NHS evidence search results were not filed in correct update group. Added to drug then realised possibly wrong location. Manually sorted obvious side-effect results into the Side-effects update set however the numbers in these grops may not match the figures from the download listed above****
