# Supplementary material for: Strategies to self-manage side-effects of adjuvant endocrine therapy among breast cancer survivors: an umbrella review of empirical evidence and clinical guidelines
Source: J Cancer Surviv. 2021 Oct 18;16(6):1296–338. doi: 10.1007/s11764-021-01114-7 (PMC9630394; doi:10.1007/s11764-021-01114-7)
Supplement: Supplementary file 3 — Supplementary file3 (DOCX 33 kb) [file 11764_2021_1114_MOESM3_ESM.docx]

| Table S1. Reviewer 1 quality assessments of systematic reviews using AMSTAR-2 checklist, with agreed consensus of overall quality | | | | | | | | | | | | | | | | | | | | | | | | | | | | | | | | | |
| --- | --- | --- | --- | --- | --- | --- | --- | --- | --- | --- | --- | --- | --- | --- | --- | --- | --- | --- | --- | --- | --- | --- | --- | --- | --- | --- | --- | --- | --- | --- | --- | --- | --- |
|  | Bae 2015 [31] | Chao 2009 [32] | Chen 2017 [40] | Chien 2015 [41] | Dowling 2017 [28] | Fritz 2013 [42] | Fritz 2014 [43] | Halsey 2015 [27] | Johns 2016 [53] | Kassab 2009 [54] | Nahm 2018 [47] | Pan 2015 [55] | Pan 2018 [44] | Roberts 2017 [33] | Salehi 2016 [34] | Yang 2017 [48] | Garcia 2015 [45] | Lee 2009 [46] | Rada 2010 [56] | Tremblay 2008 [35] | Cramer 2017 [57] | Finnegan John 2013 [36] | Taylor 2011 [37] | Cramp 2012 [58] | Bordeleau 2007 [29] | Mazzarello 2015 [38] | Ruan 2019 [30] | Lu 2020 [49] | Boing 2020 [50] | Li 2016 [51] | Roberts 2020 [59] | Chan 2020 [39] | Liu 2020 [52] |
| Did the research questions and inclusion criteria for the review include the components of PICO? | N | N | N | N | N | N | Y | N | Y | Y | N | Y | Y | N | Y | Y | Y | Y | Y | N | Y | Y | N | Y | N | Y | N | Y | N | Y | Y | N | Y |
| Did the review contain an explicit statement that the review methods were established prior to the conduct of the review and did the report justify any significant protocol deviations? | N | N | PY | N | N | N | N | N | N | Y | N | N | N | N | N | N | N | N | Y | N | Y | N | N | Y | N | N | N | N | Y | N | Y | N | N |
| Did the review authors explain their selection of the study designs for inclusion in the review? | N | N | N | Y | N | Y | N | N | Y | N | N | N | N | Y | N | N | N | N | N | Y | N | N | Y | N | N | N | N | N | N | N | N | N | N |
| Did the review authors use a comprehensive literature search strategy? | N | PY | Y | Y | PY | N | PY | N | PY | Y | N | PY | N | N | N | N | N | PY | Y | N | Y | N | PY | Y | N | PY | N | N | N | N | Y | N | Y |
| Did the authors perform study selection in duplicate? | Y | Y | Y | Y | Y | N | N | N | Y | Y | N | Y | Y | N | N | Y | Y | Y | Y | Y | Y | N | Y | Y | N | Y | N | Y | Y | Y | Y | Y | Y |
| Did the authors perform data extraction in duplicate? | N | N | Y | N | N | Y | N | N | N | Y | N | N | Y | N | Y | N | N | Y | Y | Y | Y | Y | N | Y | N | Y | N | Y | Y | Y | Y | Y | Y |
| Did the review authors provide a list of excluded studies and justify the exclusions? | N | N | N | N | N | N | N | N | N | Y | N | N | N | N | N | N | N | N | Y | N | Y | N | N | Y | N | N | N | N | N | N | Y | N | N |
| Did the review authors describe the included studies in adequate detail? | N | N | N | PY | N | N | PY | N | Y | Y | N | PY | PY | N | N | N | PY | PY | Y | PY | Y | PY | PY | PY | N | PY | N | PY | PY | N | Y | N | PY |
| Did the review authors use a satisfactory technique for assessing the risk of bias in individual studies that were included in the review? RCTs | Y | PY | Y | PY | N | Y | Y | Y | Y | Y | Y | Y | Y | PY | PY | PY | Y | PY | Y | N | Y | PY | N | Y | N | Y | N | Y | NA | NA | Y | N | N |
| Did the review authors use a satisfactory technique for assessing the risk of bias in individual studies that were included in the review? Non-randomised studies | NA | N | NA | NA | N | N | Y | NA | NA | NA | N | NA | NA | Y | NA | PY | NA | NA | NA | N | NA | NA | N | NA | NA | NA | N | NA | PY | NA | NA | NA | NA |
| Did the review authors report on the sources of funding for the studies included in the review? | N | N | N | N | N | N | N | N | N | N | N | N | N | N | N | N | N | N | N | N | N | N | N | N | N | N | N | N | N | N | N | N | N |
| If meta-analysis was performed did the review authors use appropriate methods for statistical combination of results? RCTs | NA | NA | Y | N | NA | NA | NA | NA | NA | NA | NA | Y | Y | Y | Y | N | NA | N | NA | NA | Y | NA | NA | Y | NA | NA | NA | N | N | N | Y | NA | Y |
| If meta-analysis was performed did the review authors use appropriate methods for statistical combination of results? NRSI | NA | NA | NA | NA | NA | NA | NA | NA | NA | NA | NA | NA | NA | NA | NA | N | NA | NA | NA | NA | NA | NA | NA | NA | NA | NA | NA | NA | N | NA | NA | NA | NA |
| If meta-analysis was performed, did the review authors assess the potential impact of risk of bias in individual studies on the results of the meta-analysis or other evidence synthesis? | NA | NA | N | N | NA | NA | NA | NA | NA | NA | NA | N | N | N | Y | Y | NA | N | NA | NA | Y | NA | NA | N | NA | NA | NA | Y | N | Y | Y | NA | N |
| Did the review authors account for risk of bias in individual studies when interpreting/ discussing the results of the review? | N | Y | N | N | N | N | Y | N | N | Y | Y | Y | N | Y | Y | Y | Y | Y | Y | Y | Y | Y | N | Y | N | N | N | NA | Y | Y | Y | N | N |
| Did the review authors provide a satisfactory explanation for, and discussion of, any heterogeneity observed in the results of the review? | N | Y | N | N | N | N | Y | N | N | Y | N | Y | Y | Y | Y | Y | Y | Y | Y | Y | Y | N | N | Y | N | N | N | Y | Y | Y | Y | N | Y |
| If they performed quantitative synthesis did the review authors carry out an adequate investigation of publication bias (small study bias) and discuss its likely impact on the results of the review? | NA | NA | N | N | NA | NA | NA | NA | NA | NA | NA | Y | N | N | N | Y | NA | N | NA | NA | Y | NA | NA | N | NA | NA | NA | N | N | N | Y | N | Y |
| Did the review authors report any potential sources of conflict of interest, including any funding they received for conducting the review? | Y | N | Y | Y | Y | Y | Y | Y | Y | Y | Y | Y | Y | N | Y | N | Y | N | Y | Y | Y | Y | Y | Y | N | Y | N | Y | Y | Y | Y | Y | y |
|  |  |  |  |  |  |  |  |  |  |  |  |  |  |  |  |  |  |  |  |  |  |  |  |  |  |  |  |  |  |  |  |  |  |
| Overall confidence (agreed consensus) | L | L | M | M | CL | M | M | CL | H | H | M | H | M | L | L | M | M | M | H | L | H | L | L | H | CL | L | CL | M | M | M | H | L | M |

| Table S2. Reviewer 2 quality assessments of systematic reviews using AMSTAR-2 checklist, with agreed consensus of overall quality | | | | | | | | | | | | | | | | | | | | | | | | | | | | | | | | | |
| --- | --- | --- | --- | --- | --- | --- | --- | --- | --- | --- | --- | --- | --- | --- | --- | --- | --- | --- | --- | --- | --- | --- | --- | --- | --- | --- | --- | --- | --- | --- | --- | --- | --- |
|  | Bae 2015 [31] | Chao 2009 [32] | Chen 2017 [40] | Chien 2015 [41] | Dowling 2017 [28] | Fritz 2013 [42] | Fritz 2014 [43] | Halsey 2015 [27] | Johns 2016 [53] | Kassab 2009 [54] | Nahm 2018 [47] | Pan 2015 [55] | Pan 2018 [44] | Roberts 2017 [33] | Salehi 2016 [34] | Yang 2017 [48] | Garcia 2015 [45] | Lee 2009 [46] | Rada 2010 [56] | Tremblay 2008 [35] | Cramer 2017 [57] | Finnegan John 2013 [36] | Taylor 2011 [37] | Cramp 2012 [58] | Bordeleau 2007 [29] | Mazzarello 2015 [38] | Ruan 2019 [30] | Lu 2020 [49] | Boing 2020 [50] | Li 2016 [51] | Roberts 2020 [59] | Chan 2020 [39] | Liu 2020 [52] |
| Did the research questions and inclusion criteria for the review include the components of PICO? | N | N | N | N | N | N | N | N | N | Y | N | N | N | N | N | N | N | N | N | N | N | N | N | N | N | N | N | N | N | N | N | N | N |
| Did the review contain an explicit statement that the review methods were established prior to the conduct of the review and did the report justify any significant protocol deviations? | N | N | N | N | N | N | N | N | N | N | N | N | N | N | N | N | N | N | Y | N | P Y | N | N | P Y | N | N | N | N | Y | N | Y | N | N |
| Did the review authors explain their selection of the study designs for inclusion in the review? | N | N | N | Y | N | N | Y | N | N | N | N | N | N | Y | N | N | N | N | N | Y | N | N | Y | N | N | N | N | N | Y | N | N | N | Y |
| Did the review authors use a comprehensive literature search strategy? | N | P Y | Y | P Y | N | N | Y? | N | P Y? | Y | P Y | P Y? | N | N | N | N | P Y | P Y | Y | N | P Y | N | P Y | Y | N | N | N | N | N | N | Y | N | Y |
| Did the authors perform study selection in duplicate? | Y | Y | N | Y | Y | N | N | N | Y | Y | N | Y | Y | N | N | Y | Y | Y | Y | N | Y | N | Y | Y | N | Y | N | Y | Y | Y | Y | Y | Y |
| Did the authors perform data extraction in duplicate? | N | N | Y | N | N | Y | Y | N | Y | Y | N | N | Y | N | Y | N | N | Y | Y | Y | Y | Y | N | Y | N | Y | N | Y | Y | Y | Y | Y | Y |
| Did the review authors provide a list of excluded studies and justify the exclusions? | N | N | N | N | N | N | N | N | N | N | N | N | N | N | N | N | N | N | Y | N | Y | N | N | Y | N | N | N | N | Y | N | Y | N | N |
| Did the review authors describe the included studies in adequate detail? | N | N | N | P Y | N | N | P Y | N | P Y | P Y | P Y | P Y | P Y | P Y | N | P Y | P Y | P Y | Y | P Y | Y | N | N | P Y | P Y | P Y | PY | PY | PY | PY | Y | PY | PY |
| Did the review authors use a satisfactory technique for assessing the risk of bias in individual studies that were included in the review? RCTs | Y | P Y | Y | P Y | N | Y | Y | Y | Y | Y | Y | Y | Y | P Y | N | N | Y | N | Y | N? | Y | N | N | Y | N | Y | N | Y | Y | Y | Y | PY | Y |
| Did the review authors use a satisfactory technique for assessing the risk of bias in individual studies that were included in the review? Nn-randomised studies | NA | N | NA | NA | N | N | P Y | NA | NA | NA | N | NA | NA | P Y | N | P Y | NA | NA | NA | N | NA | N | N | NA | N | NA | N | Y | Y | NA | NA | NA | NA |
| Did the review authors report on the sources of funding for the studies included in the review? | N | N | N | N | N | N | N | N | N | N | N | N | N | N | N | N | N | N | N | N | N | N | N | N | N | N | N | N | N | N | Y | N | N |
| If meta-analysis was performed did the review authors use appropriate methods for statistical combination of results? RCTs | NA | NA | NA | Y | NA | NA | NA | NA | NA | NA | NA | N | Y | N | N | Y | NA | N | NA | NA | Y | NA | NA | Y | NA | NA | NA | Y | Y | Y | Y | NA | Y |
| If meta-analysis was performed did the review authors use appropriate methods for statistical combination of results? NRSI | NA | NA | NA | NA | NA | NA | NA | NA | NA | NA | NA | NA | NA | NA | N | N | NA | NA | NA | NA | NA | NA | NA | NA | NA | NA | NA | N | N | NA | NA | NA | NA |
| If meta-analysis was performed, did the review authors assess the potential impact of risk of bias in individual studies on the results of the meta-analysis or other evidence synthesis? | NA | NA | NA | Y | NA | NA | NA | NA | NA | NA | NA | N | N | N | N | Y | NA | Y | NA | NA | Y | NA | NA | N | NA | NA | NA | N | N | Y | Y | NA | N |
| Did the review authors account for risk of bias in individual studies when interpreting/ discussing the results of the review? | Y | Y | N | Y | N | Y | Y | Y | N | Y | Y | Y | N | Y | Y | Y | Y | Y | Y | Y | Y | Y | Y | N | N | Y | N | N | Y | Y | Y | Y | N |
| Did the review authors provide a satisfactory explanation for, and discussion of, any heterogeneity observed in the results of the review? | N | Y | N | N | N | Y | Y | Y | N | Y | Y | N | Y | Y | Y | Y | Y | Y | Y | Y | Y | Y | Y | N | N | Y | Y | Y | Y | N | Y | Y | Y |
| If they performed quantitative synthesis did the review authors carry out an adequate investigation of publication bias (small study bias) and discuss its likely impact on the results of the review? | NA | NA | NA | N | NA | NA | NA | NA | NA | NA | NA | Y | N | N | N | Y | NA | N | NA | NA | Y | NA | NA | N | N | NA | NA | N | N | N | Y | NA | N |
| Did the review authors report any potential sources of conflict of interest, including any funding they received for conducting the review? | Y | N | Y | Y | Y | Y | N | Y | Y | Y | Y | Y | Y | N | Y | Y | Y | N | Y | Y | Y | Y | Y | Y | N | Y | Y | Y | N | Y | Y | Y | Y |
| Overall confidence (agreed consensus) | L | L | M | M | CL | M | M | CL | H | H | M | H | M | L | L | M | M | M | H | L | H | L | L | H | CL | L | CL | M | M | M | H | L | M |

Note: Y=Yes, N=No, PY=Partial yes, CL=critically low, L=Low, M=Moderate, H=High
